# Supplementary material for: CROP: a retromer‐PROPPIN complex mediating membrane fission in the endo‐lysosomal system
Source: EMBO J. 2022 Apr 25;41(10):e109646. doi: 10.15252/embj.2021109646 (PMC9108610; doi:10.15252/embj.2021109646)
Supplement: Supplementary file 1 — Appendix [file EMBJ-41-e109646-s002.pdf]

Appendix

Table of Contents

APPENDIX TABLE S1: ATG18 INTERACTORS IDENTIFIED IN THE SILAC APPROACH 2

APPENDIX TABLE S2: YEAST STRAINS USED IN THIS STUDY 3

APPENDIX TABLE S3: PLASMIDS USED IN THIS STUDY 5

APPENDIX TABLE S4: PRIMERS USED IN THIS STUDY 7

APPENDIX TABLE S5: COMPOUNDS USED IN THE SILAC EXPERIMENT 10

APPENDIX FIG. S1: EXPRESSION OF VPS26^{EGFP} AND VPS35^{EGFP} IS NOT AFFECTED BY WIPI1 VARIANTS 11

Appendix Table S1: Atg18 interactors identified in the SILAC approach

**Gene Name**	**Protein Name**	**Log2 Ratio (Standard media/Control)**	**Log2 Ratio (Hyperosmotic Shock/Control)**	**Log2 Ratio (Hyperosmotic shock/Standard media)**
ATG18	Autophagy-related protein 18	8.1	8.3	0.06
ATG2	Autophagy-related protein 2	5.3	4.9	-0.40
BUB1	Checkpoint Serine/Threonine-protein kinase Bub1	4.9	4.5	-0.34
SAP155	SIT4-associated protein Sap155	3.5	4.2	0.56
GIS3	Protein Gis3	1.6	2.7	0.97
SIT4	Serine/threonine-protein phosphatase PP1-1	1.8	2.7	0.74
CDC55	Protein phosphatase PP2A regulatory subunit B	1.1	2.0	1.04
VPS35	Vacuolar protein sorting-associated protein 35	1.1	1.6	0.60
VPS29	Vacuolar protein sorting-associated protein 29	0.8	1.5	0.53
VPS26	Carboxypeptidase Y-deficient protein 8	0.8	1.4	0.60
GIS2	Zinc finger protein Gis2	0.5	1.2	0.68
ILV3	Dihydroxy-acid dehydratase, mitochondrial	0.5	1.0	0.45

Appendix Table S2: Yeast strains used in this study

Strain	*Genotype*	*Source*
**BJ3505**	*MATa pep4::HIS3 prb1-Δ1.6R lys2-208 trp1-Δ101 ura3-52 gal2 can1*	*Jones et al., 1982*
**SEY6210**	*MATα leu2-3,112 ura3-52 his3-Δ200 trp1-Δ901 suc2-Δ9 lys2-801, GAL*	*Robinson et al., 1988*
**SEY6211**	*MATa leu2-3,112 ura3-52 his3-Δ200 trp1-Δ901 ade2-101 suc2-Δ9, GAL*	*Robinson et al., 1988*
**TC14**	*BJ3505 ATG18-Gly*$_6$*-FLAG*$_3$*::kanMX4, promNOP1-CAN1::URA3, arg4::natNT2*	*This study*
**TC22**	*BJ3505 promNOP1-CAN1::URA3, arg4Δ::natNT2*	*This study*
**AM3739**	*SEY6210 atg18Δ::natNT2, atg21Δ::kanMX4::loxed, hsv2Δ::kanMX4::loxed*	*This study*
**AM3740**	*SEY6210 atg18Δ::natNT2, atg21Δ::kanMX4::loxed, hsv2Δ::kanMX4::loxed, Vps5-yomCherry::kanMX4*	*This study*
**AM3741**	*SEY6210 atg18Δ::natNT2, atg21Δ::kanMX4::loxed, hsv2Δ::kanMX4::loxed, Vps17-yomCherry::kanMX4*	*This study*
**AM3743**	*SEY6210 atg18Δ::natNT2, atg21Δ::kanMX4::loxed, hsv2Δ::kanMX4::loxed, Vps26-yomCherry::kanMX4*	*This study*
**AM3745**	*SEY6210 atg18Δ::natNT2, atg21Δ::kanMX4::loxed, hsv2Δ::kanMX4::loxed Vps29-yomCherry::kanMX4*	*This study*
**AM3746**	*SEY6210 atg18Δ::natNT2, atg21Δ::kanMX4::loxed, hsv2Δ::kanMX4::loxed Vps35-yomCherry::kanMX4*	*This study*
**TC184**	*SEY6210 WT, plasmid pRS316-ATG18-HyG::URA3*	*This study*
**TC185**	*SEY6210 Vps35-yomCherry::SpHIS5, plasmid pRS316-ATG18-HyG::URA3*	*This study*
**TC186**	*SEY6210 Vps35-yomCherry::SpHIS5, vps26Δ::natNT2, plasmid pRS316-ATG18-HyG::URA3*	*This study*
**AM3743**	*SEY6210 atg18Δ:: natNT2, atg21Δ::kanMX4::loxed , hsv2Δ::kanMX4::loxed, Vps26-yomCherry::kanMX4*	*This study*
**TC181**	*SEY6210 vps26-mcherry::SpHIS5, plasmid pRS316-ATG18-HyG::URA3*	*This study*
**TC182**	*SEY6210 vps26-mcherry::SpHIS5 , vps29Δ::natNT2, plasmid pRS316-ATG18-HyG::URA3*	*This study*
**TC183**	*SEY6210 VPS26-mCherry::SpHIS5 vps35Δ::natNT2, plasmid pRS316-ATG18-HyG::URA3*	*This study*
**TC48**	*SEY6210, ATG18-yEGFP::CaURA3*	*This study*

**TC102**	*SEY6210, ATG18-yEGFP::CaURA3 vps5Δ::natNT2*	*This study*
**TC103**	*SEY6210, ATG18-yEGFP::CaURA3 vps17Δ::natNT2*	*This study*
**TC104**	*SEY6210, ATG18-yEGFP::CaURA3 vps26Δ::natNT2*	*This study*
**TC105**	*SEY6210, ATG18-yEGFP::CaURA3 vps29Δ::natNT2*	*This study*
**TC106**	*SEY6210, ATG18-yEGFP::CaURA3 vps35Δ::natNT2*	*This study*
**TC97**	*SEY6210 atg18Δ::natNT2, atg21Δ::kanMX4::loxed*	*This study*
**AM3863**	*SEY6210 vps26Δ::natNT2*	*This study*
**AM4134**	*SEY6211 vps17Δ::natNT2*	*This study*
**TC232**	*SEY6210 vps26Δ::natNT2, vps17Δ::kanMX4*	*This study*
**AM4201**	*SEY6210 atg18Δ::natNT2, atg21Δ::kanMX4::loxed, vps17Δ::kanMX4*	*This study*
**AM3774**	*SEY6210 VPS26-yomCherry::SpHIS5*	*This study*
**AM4135**	*SEY6211 vps17Δ::natNT2, VPS26-yomCherry::SpHIS5*	*This study*
**CUY9932**	*CUY100, promVPS26::HIS3-promGAL1, promVPS29::natNT2-promGAL1::VPS29-GFP::kanMX4, promVPS35::hphNT1, VPS26::TAP-URA3, vps5Δ::TRP1*	*Purushothaman et al., 2017*
**NG450**	*SEY6210 snx3Δ::kanMX4*	*This study*

Appendix Table S3: Plasmids used in this study

Plasmid	*Description*	*Source*
**A2096**	*pRS406-promNOP1::CaURA3*	*This study*
**pTC35**	*pRS316-promATG18-codATG18 ^{WT} - HA$_3$yEGFP::CaURA3*	*This study. Substitution of GFP to yEGFP using template from Obara et al., 2008,*
**pNG76**	*pRS316-promATG18-codATG18 ^{FGGG} - HA$_3$GFP::CaURA3*	*Gopaldass et al., 2017*
**pTC90**	*pRS316-promATG18-codATG18 ^{S55A} - HA$_3$yEGFP::CaURA3*	*This study*
**pTC91**	*pRS316-promATG18-codATG18 ^{S55E} - HA$_3$yEGFP::CaURA3*	*This study*
**pTC95**	*pRS316-promATG18-codATG18 ^{T56A} - HA$_3$yEGFP::CaURA3*	*This study*
**pTC96**	*pRS316-promATG18-codATG18 ^{T56E} - HA$_3$yEGFP::CaURA3*	*This study*
**pTC97**	*pRS316-promATG18-codATG18 ^{S57A} - HA$_3$yEGFP::CaURA3*	*This study*
**pTC98**	*pRS316-promATG18-codATG18 ^{S57E} - HA$_3$yEGFP::CaURA3*	*This study*
**pNG65**	*pEXP5-NT/TOPO (Invitrogen) – codATG18 ^{WT}*	*This study*
**pTC109**	*pEXP5-NT/TOPO (Invitrogen) – codATG18 ^{FGGG}*	*This study*
**pTC110**	*pEXP5-NT/TOPO (Invitrogen) – codATG18 ^{T56E}*	*This study*
**pMGDL53**	*pAR31CD-mCherry-WIPI1 WT*	*This study*
**pMGDL4**	*pAR31CD-EGFP-WIPI1 WT*	*from Tassula Proikas-Cezanne, Tübingen, Germany*
**pMGDL37**	*pAR31CD-mCherry-WIPI1 S69A*	*This study*
**pMGDL38**	*pAR31CD-EGFP-WIPI1 S69A*	*This study*
**pMGDL35**	*pAR31CD-mCherry-WIPI1 S69E*	*This study*

**pMGDL36**	*pAR31CD-EGFP-WIPI1 S69E*	*This study*
Addgene 49201	*pAC-mCherry-RAB5*	*This study*
**pMGDL55**	*pcDNA3-EGFP-VPS26*	*This study*
**pMGDL52**	*EGFP-VPS35*	*from Peter J. Cullen, Bristol, UK.*
**pMGDL70**	pLKO WIPI1-HA	*This study*

Appendix Table S4: Primers used in this study

**Primers**	***Sequence 5' -> 3'***
CAN1 cloning Fw	*TAA GCA GGA TCC ATG ACA AAT TCA AAA GAA GAC GCC GAC*
CAN1 cloning Rv	*TGC TTA CTC GAG CTA TGC TAC AAC ATT CCA AAA TTT GTC CCA AAA*
pNOP1 CHK Fw	*ATT GAG TCA TCA GCC TCT TC*
CAN1 CHK Rv	*TCC TCT ATG TCG GCG TCT TC*
arg4 KO Fw	*GAA GAG CTC AAA AGC AGG TAA CTA TAT AAC AAG ACT AAG GCA AAC CAG CTG AAG CTT CGT ACG C*
arg4 KO Rv	*AAG TAC CAG ACC TGA TGA AAT TCT TGC GCA TAA CGT CGC CAT CTG GCA TAG GCC ACT AGT GGA TCT G*
SphI-yEGFP-NotI Fw	*ATA CAT GCAT GCAT GTC TAA AGG TGA AGA ATT ATT CAC TGG TGT TG*
SphI-yEGFP-NotI Rv	*ATA AGA ATG CGG CCG CTT ATT TGT ACA ATT CAT CCA TAC CAT GGG TAA TAC CA*
atg18 KO Fw	*CAG TTA ACT CTG TAT CCT TTT CTT CTT CGG CCT GAC AAT GCG TAC GCT GCA GGT CGA C*
atg18 KO Rv	*TGT GAC GTA CGG AAG GCA GCG CGA GAC ACT TCC GTG ATC AAT CGA TGA ATT CGA GCT CG*
atg21 KO Fw	*ACT CCT TTG GAT TTG AAA TAG ACA GAT AGA AAA GGA TAT GCG TAC GCT GCA GGT CGA C*
atg21 KO Rv	*CAA TAT CTA TTA AGA TTA TGA AAA CTG CAC ATA TGC ATT AAT CGA TGA ATT CGA GCT CG*
hsv2 KO Fw	*CTG GAA AGG CAG CGA TTA TTA GAG GAC AAC TAT AAG CAT ACA TAA CTA GCA GAT GCG TAC GCT GCA GGT CGA C*
hsv2 KO Rv	*TTG TAC GTA AAT GCA CAC TTT CTC TAT ACA TAT ATA TAT ATT TAT ATT CAT GTT AAT CGA TGA ATT CGA GCT CG*
ATG18-Cterm FLAG Fw	*GGC GGC GAT TGC TTA ATA TTG TCA CAG TAT TCC ATC TTG ATG GAT GGG GGA GGC GGG GGT GGA*
ATG18-Cterm FLAG Rv	*GTA TGC GTT GTG ACG TAC GGA AGG CAG CGC GAG ACA CTT CCG TGA GAA TTC GAG CTC GTT TAA AC*
VPS5 Cterm pKT Fw	*ATG CAT CGA GCT TTG GGA GAC ATT CTA CCA AAC CAA TCT TGG TGA CGG TGC TGG TTT A*
VPS5 Cterm pKT Rv	*AGG AAC GTG ACA CAT AAA GTT ATT GTA TAC AGA TCA TCT ATC GAT GAA TTC GAG CTC G*
VPS17 Cterm pKT Fw	*ACT GAA TGC GCG CCA TGC TGC TTC ACT TTT GGG CAT GTC CAC TAA AGG TGA CGG TGC TGG TTT A*
VPS17 Cterm pKT Rv	*GAT CAC CTT GTT CAA AGG TAT GAA TTT TCT ACT TTA TAT ACG TAT CGA TGA ATT CGA GCT CG*
VPS26 Cterm pKT Fw	*ATA TTT TAA ACA ATC AGA AAT AAC ATT GTA CAG GAC CCG GGG TGA CGG TGC TGG TTT A*
VPS26 Cterm pKT Rv	*AGA ACC ACA TCT TCA CCT TAT TTA AGG TCG AGC TTT TCT ATC GAT GAA TTC GAG CTC G*

VPS29 Cterm pKT Fw	*TGG AGA AGT GAA GGT CGA TAA AGT GGT TTA TGA AAA GGA AGG TGA CGG TGC TGG TTT A*
VPS29 Cterm pKT Rv	*GAC ATC ATA GAA ATG CAT AAA AAT GAA AAT GGC TAC CCT ATC GATG AAT TCG AGC TCG*
VPS35 Cterm pKT Fw	*GAA AGT CAA AGA GAA GTT GAC GAT CGT TTC AAA GTC ATA TAT GTA GGT GAC GGT GCT GGT TTA*
VPS35 Cterm pKT Rv	*TTT ATC TTG GGC ATG TAC GAA GAG CAA GTA CGT TAT TTA ATC GAT GAA TTC GAG CTC G*
vps5 KO Fw	*AGG AAC GTG ACA CAT AAA GTT ATT GTA TAC AGA TCA TCT AGC ATA GGC CAC TAG TGG ATC TG*
vps5 KO Rv	*ATT TTA TAA ACT TTC ATA CAT CCT GCA ATA ACA AGC CAT GCA GCT GAA GCT TCG TAC GC*
vps17 KO Fw	*TTG TTC AAA GGT ATG AAT TTT CTA CTT TAT ATA CGT ATT AGC ATA GGC CAC TAG TGG ATC TG*
vps17 KO Rv	*TAC TGT ACC CTT AGT CAA TCC ATC TAT CCT CTG AAC AAT GCA GCT GAA GCT TCG TAC GC*
vps26 KO Fw	*AGA ACC ACA TCT TCA CCT TAT TTA AGG TCG AGC TTT TCT AGC ATA GGC CAC TAG TGG ATC TG*
vps26 KO Rv	*ATT GTA AAA GAA TCC AAG CAC AAC TAT TAT TAG CAT TAT GCA GCT GAA GCT TCG TAC GC*
vps29 KO Fw	*GAC ATC ATA GAA ATG CAT AAA AAT GAA AAT GGC TAC CCT AGC ATA GGC CAC TAG TGG ATC TG*
vps29 KO Rv	*TAG TGG CGA AAA GGT CAT AGA ATT ATT CGC CTA AAT TAT GCA GCT GAA GCT TCG TAC GC*
vps35 KO Fw	*ATC TTG GGC ATG TAC GAA GAG CAA GTA CGT TAT TTA ACT AGC ATA GGC CAC TAG TGG ATC TG*
vps35 KO Rv	*AAG GAG GAG GAC GAG AAA GAA GAA GCT GAA AAA CAC AAT GCA GCT GAA GCT TCG TAC GC*
atg18^{S55A} Fw	*GTC GAG ATG TTG TTC GCC ACC TCG TTA CTA GCC CTC GTT GGG ATA G*
atg18^{S55A} Rv	*CTA GTA ACG AGG TGG CGA ACA ACA TCT CGA CGA TAG CAT AGC CCC C*
atg18^{S55E} dsDNA	*GCG ATC GCA ATA TTC AAT TGT GAG CCC TTC GGA AAA TTT TAT TCA GAG GAC AGT GGG GGC TAT GCT ATC GTC GAG ATG TTG TTC GAG ACC TCG TTA CTA GCC CTC GTT GGG ATA GGC GAT CAA CCT GCG CTT TCA CCA AGG AGA TTG CGT ATA ATC AAC ACA AAA AAA CAT TCT ATT ATC TGT GAG GTG ACT TTC CCT ACT TCT ATT CTG AGT GTG AAA ATG AAT AAG TCT CGA TTG GTG GTA CTT TTA CAA GAG CAG ATT TAT ATT TAT GAT ATC AAC ACC ATG AGA CTA TTG CAT ACT ATA GAA ACA AAC CCT AAC CCA CGT GGC CTT ATG GCT ATG TCT CCT TCG GTA GCC AAC AGC TAT TTA GTG TAT CCA TCA CCA CCA AAA GTT ATT AAC TCC GAA ATT AAA GCT CAT GCC ACC ACA AAC AAT ATC ACA TTG TCA GTT GGT GGC AAC ACA GAG ACC AGT TTC AAG AGA GAT CAG CAA GAT GCT GGC CAT AGT GAG GAT CC*
atg18^{T56A} Fw	*GAG ATG TTG TTC TCC GCC TCG TTA CTA GCC CTC GTT GGG ATA GGC G*

atg18^{T56A} Rv	*GGG CTA GTA ACG AGG CGG AGA ACA ACA TCT CGA CGA TAG CAT AGC C*
atg18^{T56E} *Fw*	*GAG ATG TTG TTC TCC GAA TCG TTA CTA GCC CTC GTT GGG ATA GGC GAT*
atg18^{T56E} *Rv*	*GAG GGC TAG TAA CGA TTC GGA GAA CAA CAT CTC GAC GAT AGC ATA GCC*
atg18^{S57A} *Fw*	*ATG TTG TTC TCC ACC GCG TTA CTA GCC CTC GTT GGG ATA GGC GAT C*
atg18^{S57A} *Rv*	*CGA GGG CTA GTA ACG CGG TGG AGA ACA ACA TCT CGA CGA TAG CAT A*
atg18^{S57E} *Fw*	*ATG TTG TTC TCC ACC GAG TTA CTA GCC CTC GTT GGG ATA GGC GAT CA*
atg18^{S57E} *Rv*	*ACG AGG GCT AGT AAC TCG GTG GAG AAC AAC ATC TCG ACG ATA GCA TA*
snx3 KO Fw	*TTA ATA CTG CAT AAA AGA GGA CGG CAA GAA CAG CTG AGA AGC GGT ACG CAT CTA CAC AAA GTC GTA CGC TGC AGG TCG AC*
snx3 KO Rev	*CGT AAA AGA GTT CTT TTC AGC CGA CAA ACT TTT CAG CTT CAA TGA ACC TCA CGA GAA CGC ATA GGC CAC TAG TGG ATC TG*
wipi1 S69A Fw	*CGC CTC TTC TCC GTC AGC CTG GTG GTG*
wipi1 S69A Rv	*CAC CAC CAG GCT GAC GGA GAA GAG GCG*
wipi1 S69E Fw	*CGC CTC TTC TCC GAG AGC CTG GTG GTG*
wipi1 S69E Rv	*CAC CAC CAG GCT CTC GGA GAA GAG GCG*
wipi1 CRISPR Fw	*CAC CGC TTG AAG ATG TGT ACC GTC T*
wipi1 CRISPR Rv	*AAA CAG ACG GTA CAC ATC TTC AAG C*
siRNA vps35 Fw	*CTG GAC ATA TTT ATC AAT ATA*
siRNA vps35 Rv	*TAT ATT GAT AAA TAT GTC CAG*
mCherry-WIPI1 Age1-mcherry Fw	*CTA CCG GTC GCC ACC ATG GTG AGC AAG GGC GAG GAG G*
mCherry-WIPI1 Age1-mcherry Rv	*GCT CGA GAT CTG AGT CCG GAC TTG TAC AGC TCG TCC ATG CCG*
mCherry-WIPI1 EGFP-WIPI1-EcoR1 Fw	*TCC GGA CTC AGA TCT CGA GCT ATG GAG GCC GAG GCC GCG*
mCherry-WIPI1 EGFP-WIPI1-EcoR1 Rv	*CAG AAT TCT CAT GAC TGC TTC GTT TTG CCC TTC TG*
hVPS26 Fw	*GGG AGA CCC AAG CTT GGT ACC GAG CTC GGA TCC ACT AGT AAT GAG TTT TCT TGG AGG CTT TTT TGG*
hVPS26 Rv	*CTC GAG CGG CCG CCA GTG TGA TGG ATA TCT GCA GAA TTC TCT ATG ATG ATG ATG ATG GGA TCC AC*

Appendix Table S5: Compounds used in the SILAC experiment

**Condition**	Control	WT condition	Salt shock
**Strain used**	sTC22	sTC14	sTC14
**Arginine**	R0<br>Light L-Arginine	R6<br>L-Arginine:HCl ($^{13}C6$)	R10<br>L-Arginine:HCl ($^{13}C6$, $^{15}N4$)
**Lysine**	K0<br>Light L-Lysine	K4<br>L-Lysine-2HCl (4,4,5,5-D4)	K8<br>L-Lysine-2HCl ($^{13}C6$; $^{15}N2$)

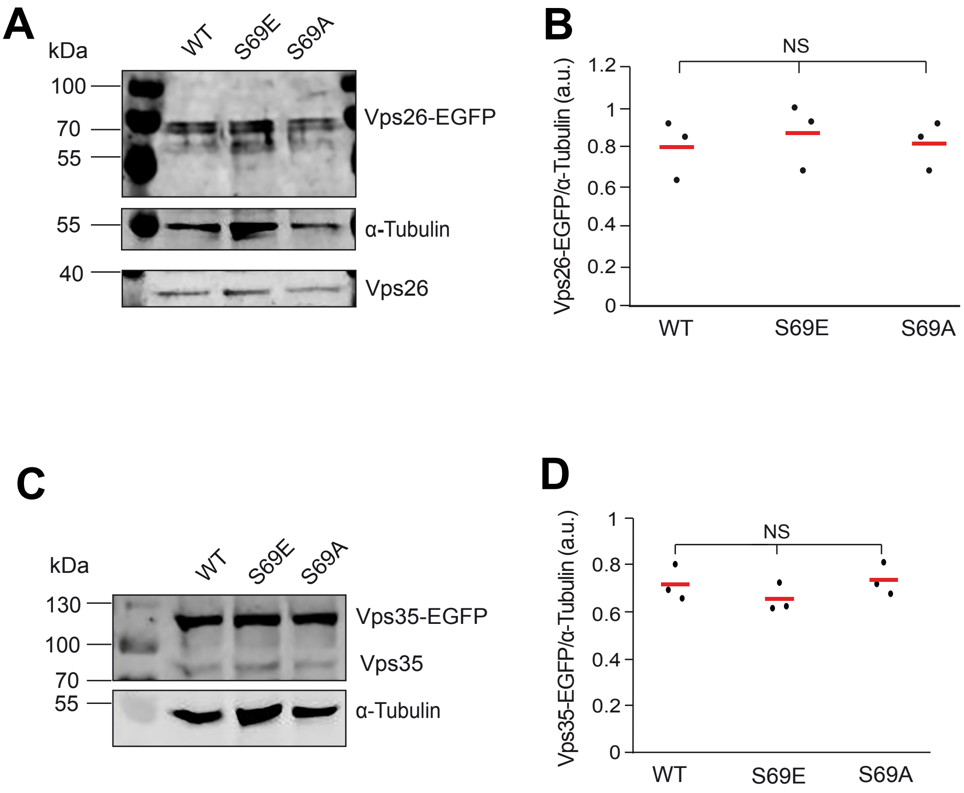

Appendix Fig. S1: Expression of Vps26^{EGFP} and Vps35^{EGFP} is not affected by WIPI1 variants

Lysates (50 µg per sample) from the cells in Figures 9A and EV6 were analyzed by SDS-PAGE and Western blot.

A Vps26^{EGFP} decorated with anti-Vps26 in lysates from Fig. 9A. Tubulin served as loading control.

B Signals from three independent experiments in A were quantified on a LICOR Odyssey fluorescence scanner. Mean values ± SD are shown. n=3 independent experiments. P values are indicated and were calculated by t-Test. The analysis was performed with 99% confidence: NS = not significant.

C Vps35^{EGFP} decorated in lysates from Fig. EV6. Tubulin served as loading control.

D Signals from three independent experiments in C were quantified. Results are presented as in B.
