# Supplementary material for: CROP: a retromer‐PROPPIN complex mediating membrane fission in the endo‐lysosomal system
Source: EMBO J. 2022 Apr 25;41(10):e109646. doi: 10.15252/embj.2021109646 (PMC9108610; doi:10.15252/embj.2021109646)
Supplement: Supplementary file 2 — Expanded View Figures PDF [file EMBJ-41-e109646-s003.pdf]

Expanded View Figures

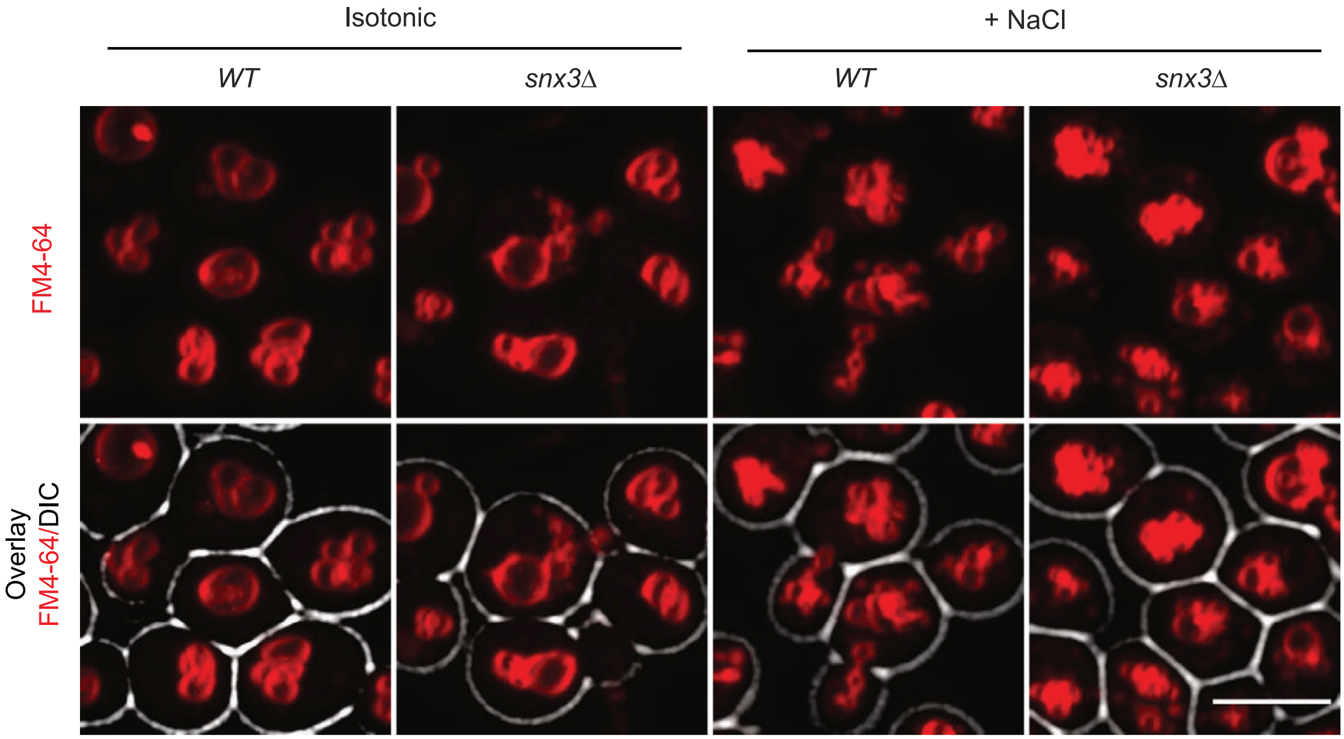

Figure EV1. Vacuole structure and vacuole fission of *snx3*Δ mutants.

Cells were logarithmically grown in YPD and stained with FM4-64. Vacuole morphology was imaged as in Fig 2A, before and after a mild salt shock with 0.5 M of NaCl for 15 min. Scale bar: 5 µm.

Figure EV2. Analysis of the functionality of Atg18^{T56E}.

A Atg18^{T56E} supports normal autophagic activity *in vivo*. Cells were logarithmically cultured in SD complete and then transferred to SD or SD-N medium for 5 h. Autophagic activity of ATG18^{WT} and ^{T56E} mutant was evaluated by measuring *pho8*Δ*60* activity (Noda & Klionsky, 2008). Error bars represent the SEM. $n = 6$ independent experiment. An unpaired *t*-test was used for statistical analysis, bars represent the mean and errors bars the SEM, $**P < 0.01$.

B Secondary structures of purified, recombinant Atg18^{WT}, and Atg18^{T56E} were analyzed using circular dichroism (CD).

C Thermal melting CD spectra of Atg18^{WT} and Atg18^{T56E}, shown in the range of 198–202 nm, where the greatest changes occurred.

D Full thermal melting spectra (198–220 nm) of Atg18^{WT} and Atg18^{T56E}, which underlie the compilation in C. Wavelengths used for C are highlighted in green.

E Lipid binding activity. Small unilamellar vesicles without phosphoinositides (99.5%PC, 0.5% PE Cy5.5), or a parallel preparation with phosphoinositides (89.5% PC + 5% PI3P, 5% PI(3,5)P_2, 0.5% PE Cy5.5) were incubated (10 min, 25°C) with Atg18^{WT} or Atg18^{T56E} (1.5 µM). The vesicles were sedimented by centrifugation and supernatants (Sup.) and pellets were analyzed by SDS–PAGE and Coomassie staining.

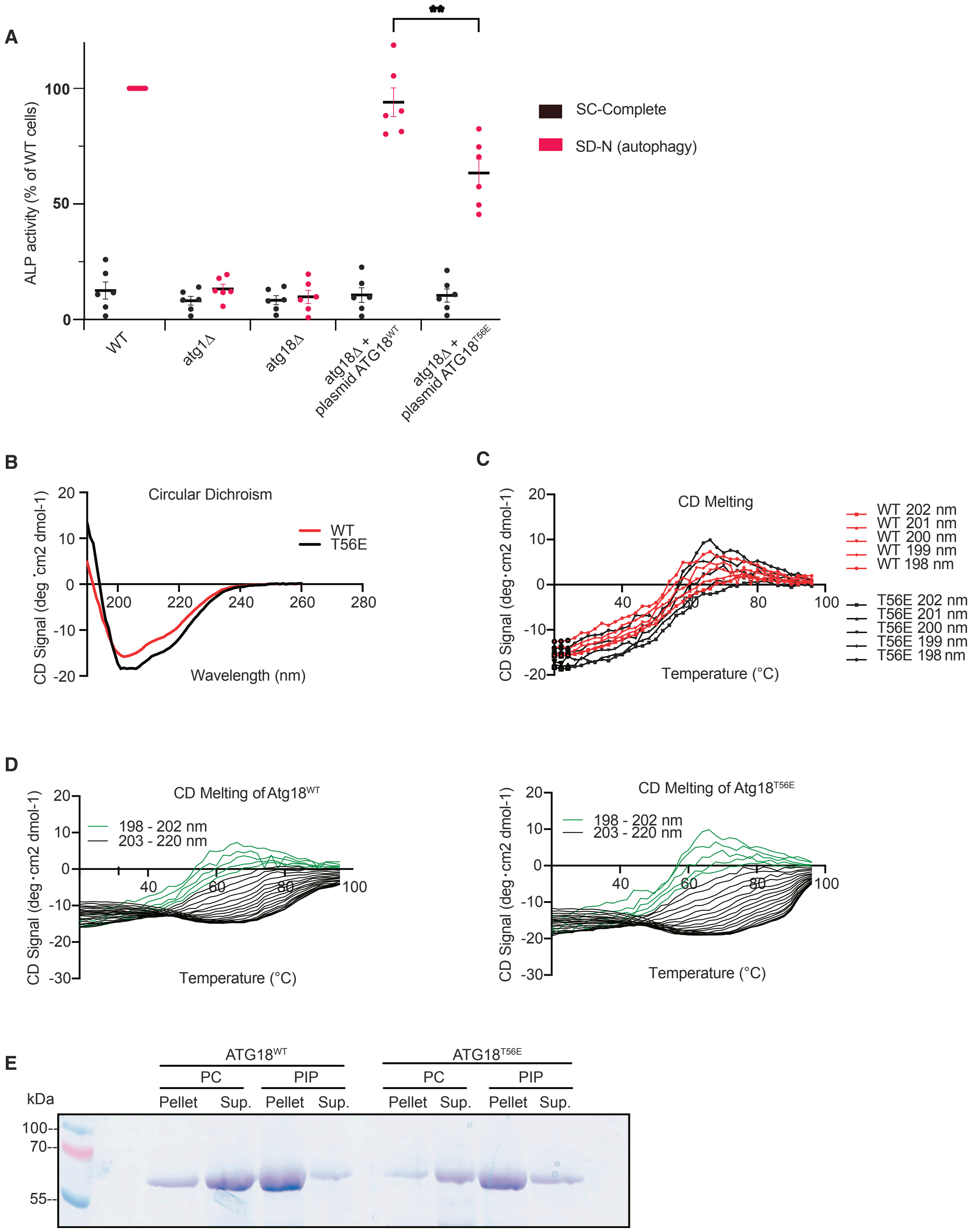

Figure EV2.

 © 2022 The Authors

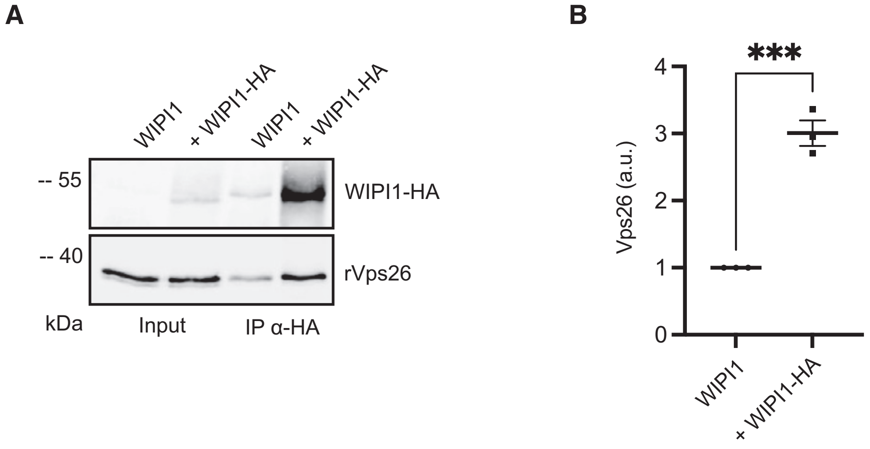

Figure EV3. Adsorption of recombinant mammalian retromer on immobilized WIPI1^{HA}.

A HK2 cells were transfected with expression constructs for WIPI1^{HA}. Cell extracts were incubated with anti-HA beads to immobilize WIPI1^{HA} and washed. The beads were incubated with recombinant human retromer purified from *E. coli*. Adsorbed proteins were analyzed by SDS–PAGE and Western blotting using the antibodies indicated in brackets.

B The intensity of the interacting hVps26 was quantified on a LICOR fluorescence imager and normalized to the signal in the sample without HA-tag (set to 1). $n = 3$ biological replicates, using an unpaired t-test. Bars represent the mean, and error bars represent the SEM, $^{**}P < 0.001$.

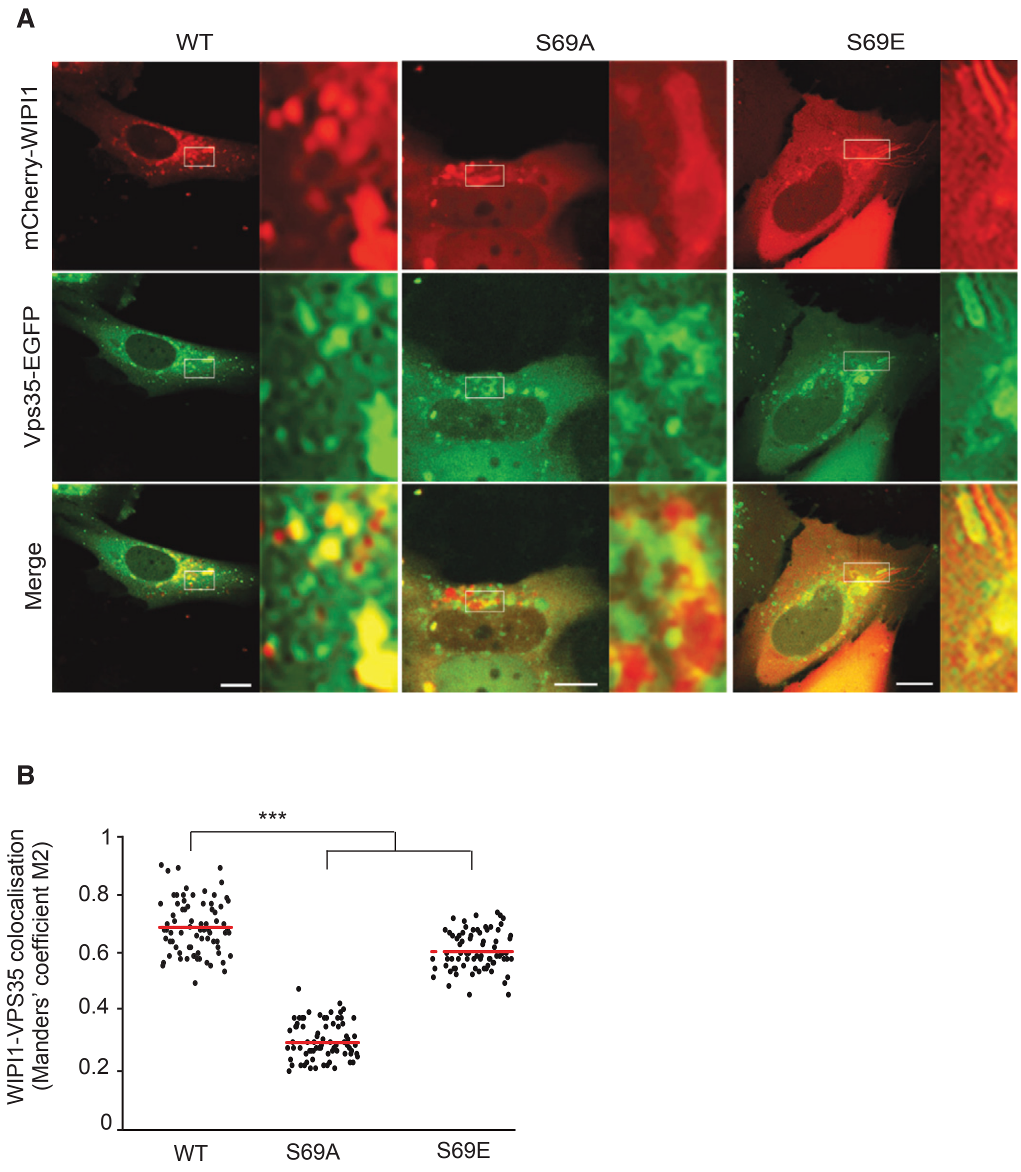

Figure EV4. Colocalization of WIPI1^{S69} variants with hVps35.

A The indicated WIPI1$^{S69\text{-}mCherry}$ variants and Vps35^{EGFP} were expressed for 18 h in HK2 cells, from which endogenous WIPI1 had been deleted. The cells were analyzed by confocal microscopy. Scale bars: 10 µm. Insets show enlargements of the outlined areas.

B Quantification of the colocalization in *a*, using Manders' coefficient M2. $N = 3$ independent experiments with a total of 195 cells were quantified per condition. *P* values were calculated by unpaired Student's *t*-test. The analysis was performed with 99% confidence: ***$P < 0.001$.

 © 2022 The Authors

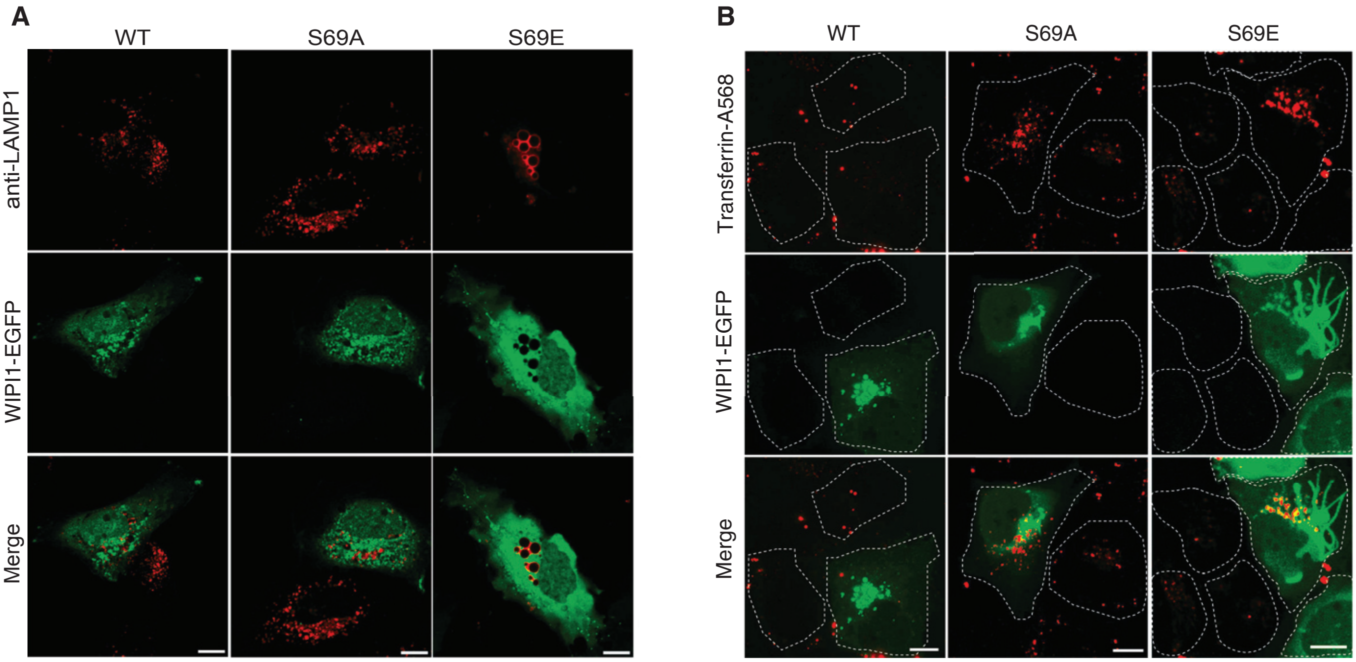

Figure EV5. Dominant negative effect of EGFP-WIPI1^{S69E} on LAMP1 compartments and on transferrin recycling.

A LAMP1 compartments. HK2 cells expressing WIPI1$^{WT\text{-}EGFP}$, WIPI1$^{S69E\text{-}EGFP}$, or WIPI1$^{S69A\text{-}EGFP}$ were fixed 18 h after transfection. The cells were stained for immunofluorescence analysis with anti-LAMP1 antibody and imaged by confocal microscopy. Scale bars: 10 μm.

B Tf recycling. HK2 cells were transfected with WIPI1$^{WT\text{-}EGFP}$, WIPI1$^{S69E\text{-}EGFP}$, or WIPI1$^{S69A\text{-}EGFP}$ for 18 h. Then, they were serum-starved for 1 h, loaded with Alexa Fluor 568-conjugated Tf, chased at 37°C for 1 h in medium without labeled Tf, and analyzed by confocal microscopy. Scale bar: 10 μM. The white dashed lines indicate the circumference of the cells.

© 2022 The Authors

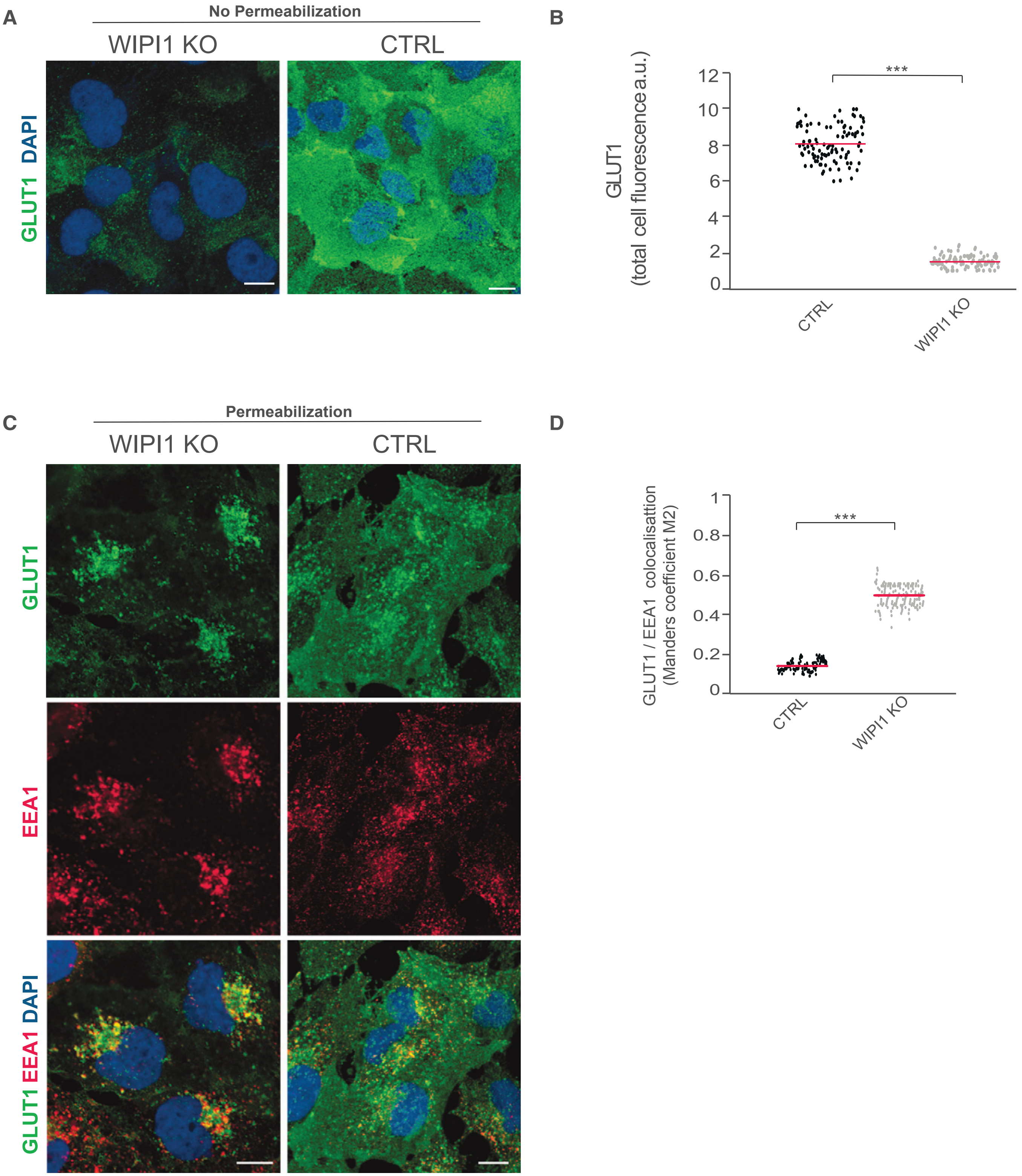
A
No Permeabilization
WIPI1 KO
CTRL
GLUT1 DAPI
B
GLUT1
(total cell fluorescence a.u.)
12
10
8
6
4
2
0
***
CTRL
WIPI1 KO
C
Permeabilization
WIPI1 KO
CTRL
GLUT1
EEA1
GLUT1 EEA1 DAPI
D
GLUT1 / EEA1 colocalisation
(Manders coefficient M2)
1
0.8
0.6
0.4
0.2
0
***
CTRL
WIPI1 KO

Figure EV6.

Figure EV6. WIPI1 promotes transport of GLUT1 to the cell surface.

A GLUT1 surface expression. Control and WIPI1 KO cells were fixed and stained with antibody to GLUT1 (green) and with DAPI (blue), without detergent permeabilization. Scale bars: 10 μm.

B Quantification of GLUT1 immunofluorescence in cells from A. Regions of interest (ROIs) corresponding to each cell and in some regions outside the cells (background) were manually defined using ImageJ software. Total cell fluorescence was integrated and corrected for background fluorescence. 150 cells per condition, stemming from three independent experiments, were analyzed. *P* values are indicated and were calculated by an unpaired Student's *t*-test. The analysis was performed with 99% confidence. $^{***}P < 0.0001$.

C Immunofluorescent staining of intracellular GLUT1 (green) and EEA1 (red) in CTRL and WIPI1KO HK2 cells. Prior to staining, the cells had been fixed and permeabilized with 0.05% saponin. Scale bars: 10 μm.

D GLUT1/EEA1 colocalization was assessed in cells from C using Manders' colocalization coefficient M2, calculated in ImageJ. The colocalization was quantified in 120 images taken from three independent experiments. *P* values were calculated by an unpaired Student's *t*-test. The analysis was performed with 99% confidence: $^{***}P < 0.0001$.

A

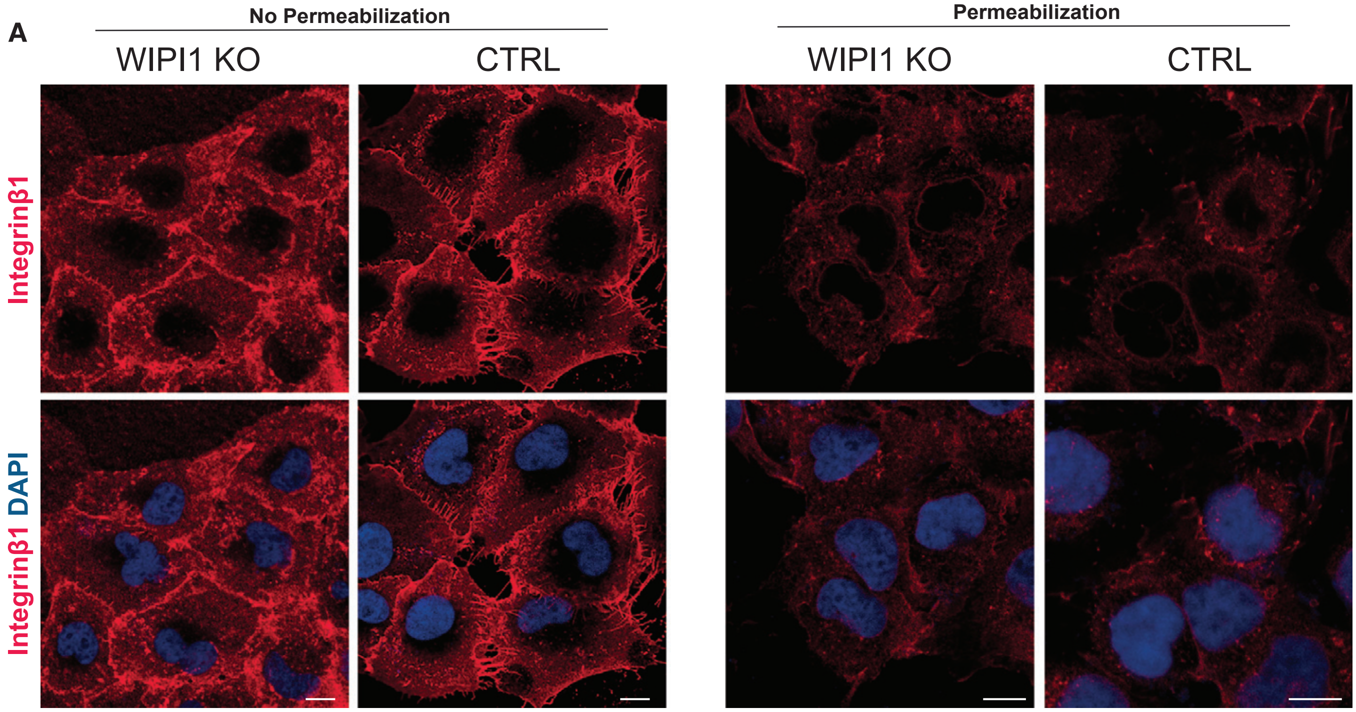

B

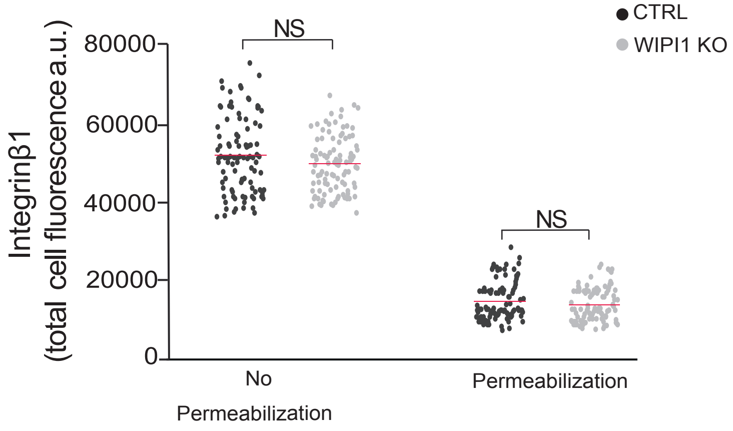

Figure EV7. Surface expression of α5β1 integrin is independent of WIPI1.

A β1 integrin surface expression. Control and WIPI1KO cells were fixed and stained with antibody to β1 integrin (red), and with DAPI (blue). Where indicated, cells had been permeabilized with saponin 0.05%) prior to staining. Scale bars: 10 μm.

B Quantification of β1 integrin-immunofluorescence in cells from A. Regions of interest (ROIs) corresponding to each cell and in some regions outside the cells (background) were manually defined using ImageJ software. Total cell fluorescence was integrated and corrected for background fluorescence. 180 cells per condition, stemming from three independent experiments, were analyzed. Red bars show the means. *P* values were calculated by unpaired Student's *t*-test. The analysis was performed with 99% confidence. NS = not significant ($P > 0.01$).

 © 2022 The Authors
