# Supplementary material for: CROP: a retromer‐PROPPIN complex mediating membrane fission in the endo‐lysosomal system
Source: EMBO J. 2022 Apr 25;41(10):e109646. doi: 10.15252/embj.2021109646 (PMC9108610; doi:10.15252/embj.2021109646)
Supplement: Supplementary file 3 [file EMBJ-41-e109646-s001.pdf]

THE EMBO JOURNAL

CROP: A Retromer-PROPPIN complex mediating membrane fission in the endo-lysosomal system

Thibault Courtellement, Maria De Leo, Navin Gopaldass, and Andreas Mayer
DOI: 10.15252/embj.2021109646

Corresponding author: Andreas Mayer (andreas.mayer@unil.ch)

Review Timeline:

Submission Date:	3rd Sep 21
Editorial Decision:	30th Sep 21
Revision Received:	4th Feb 22
Editorial Decision:	1st Mar 22
Revision Received:	21st Mar 22
Accepted:	24th Mar 22

Editor: David del Alamo

Transaction Report:

(Note: With the exception of the correction of typographical or spelling errors that could be a source of ambiguity, letters and reports are not edited. Depending on transfer agreements, referee reports obtained elsewhere may or may not be included in this compilation. Referee reports are anonymous unless the Referee chooses to sign their reports.)

1st Editorial Decision 30th Sep 2021

Dear Prof. Mayer,

Thank you again for the submission of your manuscript entitled "CROP: A Retromer-PROPPIN complex mediating membrane fission in the endo-lysosomal system" and for your patience during the review process. We have now received the reports from the referees, which I copy below.

As you can see from their comments, all three referees are supportive of your work, but point out to several significant experimental concerns that will require your attention before your manuscript can be published in The EMBO Journal.

Based on the overall interest expressed in the reports, I would like to invite you to address the comments of all referees in a revised version of the manuscript. I should add that it is The EMBO Journal policy to allow only a single major round of revision and that it is therefore important to resolve the main concerns at this stage. I believe the concerns of the referees, although probably requiring a significant amount of work, are reasonable and addressable, but we are aware that many laboratories cannot function at full efficiency during the current COVID-19/SARS-CoV-2 pandemic, so please contact me if you have any questions, need further input on the referee comments or if you anticipate any problems in addressing any of their points. Please, follow the instructions below when preparing your manuscript for resubmission.

I would also like to point out that as a matter of policy, competing manuscripts published during this period will not be taken into consideration in our assessment of the novelty presented by your study ("scooping" protection). We have extended this 'scooping protection policy' beyond the usual 3 month revision timeline to cover the period required for a full revision to address the essential experimental issues. Please contact me if you see a paper with related content published elsewhere to discuss the appropriate course of action.

When preparing your letter of response to the referees' comments, please bear in mind that this will form part of the Review Process File, and will therefore be available online to the community. For more details on our Transparent Editorial Process, please visit our website: https://www.embopress.org/page/journal/14602075/authorguide#transparentprocess

Again, please contact me at any time during revision if you need any help or have further questions.

Thank you very much again for the opportunity to consider your work for publication. I look forward to your revision.

Best regards,

David

David del Alamo, PhD.
Editor
The EMBO Journal

When submitting your revised manuscript, please carefully review the instructions below and include the following items:

1) a .docx formatted version of the manuscript text (including legends for main figures, EV figures and tables). Please make sure that the changes are highlighted to be clearly visible.

2) individual production quality figure files as .eps, .tif, .jpg (one file per figure).

3) a .docx formatted letter INCLUDING the reviewers' reports and your detailed point-by-point response to their comments. As part of the EMBO Press transparent editorial process, the point-by-point response is part of the Review Process File (RPF), which will be published alongside your paper.

4) a complete author checklist, which you can download from our author guidelines (https://wol-prod-cdn.literatumonline.com/pb-assets/embo-site/Author Checklist%20-%20EMBO%20J-1561436015657.xlsx). Please insert information in the checklist that is also reflected in the manuscript. The completed author checklist will also be part of the RPF.

5) Please note that all corresponding authors are required to supply an ORCID ID for their name upon submission of a revised manuscript.

6) We require a 'Data Availability' section after the Materials and Methods. Before submitting your revision, primary datasets produced in this study need to be deposited in an appropriate public database, and the accession numbers and database listed under 'Data Availability'. Please remember to provide a reviewer password if the datasets are not yet public (see

https://www.embopress.org/page/journal/14602075/authorguide#datadeposition). If no data deposition in external databases is needed for this paper, please then state in this section: This study includes no data deposited in external repositories. Note that the Data Availability Section is restricted to new primary data that are part of this study.

Note - All links should resolve to a page where the data can be accessed.

7) When assembling figures, please refer to our figure preparation guideline in order to ensure proper formatting and readability in print as well as on screen:
http://bit.ly/EMBOPressFigurePreparationGuideline

Please remember: Digital image enhancement is acceptable practice, as long as it accurately represents the original data and conforms to community standards. If a figure has been subjected to significant electronic manipulation, this must be noted in the figure legend or in the 'Materials and Methods' section. The editors reserve the right to request original versions of figures and the original images that were used to assemble the figure.

8) For data quantification: please specify the name of the statistical test used to generate error bars and P values, the number (n) of independent experiments (specify technical or biological replicates) underlying each data point and the test used to calculate p-values in each figure legend. The figure legends should contain a basic description of n, P and the test applied. Graphs must include a description of the bars and the error bars (s.d., s.e.m.).

9) We would also encourage you to include the source data for figure panels that show essential data. Numerical data can be provided as individual .xls or .csv files (including a tab describing the data). For 'blots' or microscopy, uncropped images should be submitted (using a zip archive or a single pdf per main figure if multiple images need to be supplied for one panel). Additional information on source data and instruction on how to label the files are available at .

10) We replaced Supplementary Information with Expanded View (EV) Figures and Tables that are collapsible/expandable online (see examples in https://www.embopress.org/doi/10.15252/embj.201695874). A maximum of 5 EV Figures can be typeset. EV Figures should be cited as 'Figure EV1, Figure EV2" etc. in the text and their respective legends should be included in the main text after the legends of regular figures.

- For the figures that you do NOT wish to display as Expanded View figures, they should be bundled together with their legends in a single PDF file called *Appendix*, which should start with a short Table of Content. Appendix figures should be referred to in the main text as: "Appendix Figure S1, Appendix Figure S2" etc. See detailed instructions regarding expanded view here: .

- Additional Tables/Datasets should be labelled and referred to as Table EV1, Dataset EV1, etc. Legends have to be provided in a separate tab in case of .xls files. Alternatively, the legend can be supplied as a separate text file (README) and zipped together with the Table/Dataset file.

11) Our journal encourages inclusion of *data citations in the reference list* to directly cite datasets that were re-used and obtained from public databases. Data citations in the article text are distinct from normal bibliographical citations and should directly link to the database records from which the data can be accessed. In the main text, data citations are formatted as follows: "Data ref: Smith et al, 2001" or "Data ref: NCBI Sequence Read Archive PRJNA342805, 2017". In the Reference list, data citations must be labeled with "[DATASET]". A data reference must provide the database name, accession number/identifiers and a resolvable link to the landing page from which the data can be accessed at the end of the reference. Additional instructions are available at .

Further information is available in our Guide For Authors: https://www.embopress.org/page/journal/14602075/authorguide

Revision to The EMBO Journal should be submitted online within 90 days, unless an extension has been requested and approved by the editor; please click on the link below to submit the revision online before 29th Dec 2021:

Link Not Available

Referee #1:

The paper by T. Courtellemont et al. proposes an interesting concept of the formation of a retromer-PROPPIN complex with a function in membrane fission at endosomes and this is poorly studied and understood. It seems rather unnecessary to give this complex a new name to try and own it. Retromer makes many complexes and they do not have separate names.
I have one major problem with this work that I believe must be addressed and a significant number of experimental issues cleared up before this paper should be considered for publication in EMBO for which it would likely be suitable if the authors' answers are acceptable.

In its early days, retromer in yeast was regarded as a heteropetamer but this is historical and not correct. On a gel of yeast retromer there is as much of the sorting nexin SNX3 homologue Grd19p as there is of SNXBARs. Yeast retromer CSC is also able to form membrane attached assemblies with just Grd19p/SNX3 alone (Ungermann and Briggs+Owen labs). Nowhere in this manuscript is Grd19p studied and its role absolutely must be because it is critical to the main message of this work in that SNX BARs compete with Proppins but do they compete with Grd19p/SNX3?
In the first place was Grd19p/SNX3 found in any SILAC experiments?
What happens to the proppin+retromer complexes if you add excess Grd19p/SNX3 rather than SNX BARS?

Lines 160-170 What happens in VPS29 and VPS35 delete cells, as the text say these are made and tested, on ATG18YFP immunoabsorption

T56E is important to this work its yield is the same according to the authors but its fold should be checked - a simple CD would suffice - solving the structure of the mutant is unnecessary

TfR is not meant to be retromer dependent in its transport and to come from early/recycling endosomes. This would suggest WIP1 is a general fusion machine not particularly specific for retromer but functioning anywhere on the endocytic system - could the authors comment please

Discussion - please could the authors explain how they envisage retromer CSC to drive tubule scission when recruited by proppins but not when recruited by SNXBARs when the methods of recruitment, since they compete with each other, would presumably be expected to be the same?

Technical questions:
Atg18 forms a complex with Vps26/29/35
As the authors pointed out, a retromer-containing coat can be extracted from yeast. However, the formation of the retromer-Vps5/Vps17 complex requires two types of protein-protein interactions: first, between unstructured N-term part of Vps5 and Vps29 (Seaman and Williams, 2002, Collins et al., 2005) and the second interaction between loops of Vps26 and BAR-domain of Vps5 that was observed in CryoEM structure of the retromer-Vps5 complex (Kovtun et al., 2018). The mass spectrometry analysis results suggest that complex assembly is affected when an Atg18-retromer complex is formed. The authors suggest competing interactions between Vps5 and Atg18 for Vps26 (and possibly Vps35), could authors suggest how Atg18 might affect the interaction between Vps5/Vps29 since they claim complex formation is completely disrupted.

Fig1C It looks like there are at least two different yomCherry -Vps5 with almost 10kDa weight difference. Could you please comment on this - why?

Atg18 and sorting nexins compete for binding to Vps26/29/35
It needs to be clarified what kind of spectroscopy or microscopy methods were used to assess the change of fluorophore properties in the following passage: "To assay formation of CROP directly, we purified Atg18 from bacteria and CSC from yeast, where the Vps29 was labeled with GFP. We noted that the addition of Atg18 to CSC-GFP induced a strong shift in the fluorescence signal of the GFP. Ligand binding can change the properties of protein-bound fluorophores via changes in orientation, contacts, or rotational freedom of the fluorophore. Such changes are a common tool used in titration experiments to determine binding constants."
In Fig. 4, positions a and b need to be swapped as b get mentioned in the text first. It is also puzzling that there is an absence of the signal for CSC-GFP when SNX dimer was added to the CSC-GFP/Atg18 complex. As the authors mentioned previously, retromer can form a stable complex with SNXs. So it would be expected that the mixture loaded into the native gel would contain combinations of different species; showing up as possibly smearing (similar to that observed for ATG18 in the green channel). It would also be essential to include CSC-GFP/SNX complex as a control.

Line 203:"Purified CSC forms two major bands, in line with previous observations of monomers and dimers (Kendall et al, 2020)."

On the gel, CSC-GFP/Atg18 mix doesn't have an upper (suggested dimer) band. Does it mean the addition of Atg18 affects dimerization of the trimer to form the arches as seen by the Briggs lab? Regardless the Briggs lab paper should be referenced

In general it is important to have a real protein ladder for SDS-gels and Western blots visible and not just random numbers added often in relatively different positions.

In Fig7a the whole image of the gel needs to be shown, possibly in supplementary, as it looks like there are some problems with the background if it was a combination of two different gels in should be clearly stated.

There are no secondary antibodies stated nor methods of visualisation
CROP is required for protein exit from mammalian endosomes

"Their abundance and size were further increased by simultaneous knockdown of hVps35" Are these tubules SNX-positive please show?
Discussion
"The current models suggest a two-layered coat, in which an inner layer of SNX proteins recruits a peripheral layer of arch-shaped CSC complexes." This statement needs to be clarified. The two-layer coat was observed only for SNX-BAR-retromer coat (Kovtun et al., 2018), in the case of SNX3-retromer coat, CSC is attached directly to the membrane with the help of cargo and PX-domain of SNX3 (Leneva et al., 2021). In both proposed models dimerization of Vps26 plays an important role, how does it fit into suggested model of interaction Vps26/Atg18? What part of Vps26 (the same loops as in case of Vps5) might be involved in interaction?
M&M
No secondary antibodies or visualisation methods are described.
No information on MW protein ladders in included.

Referee #2:

In this manuscript, Mayer and colleagues define an interaction of the CSC (Retromer) with Atg18 (WIPI)that is required for vacuole scission in yeast and scission of endosomal transport carriers in mammalian cells. They term this new complex, the CROP (acronym not defined). They further define that sorting nexin vps17 impairs the recruitment and scission activity of Atg18. Moreover, they demonstrate that a highly conserved serine residue found in both Atg18 and WIPI proteins is involved in interaction of Atg18 with the CSC, and hence, scission of the yeast-like vacuole, GUVs and mammalian endosomes. Overall, this is a very interesting paper with highly supportive data that makes an important discovery regarding the role of Atg18/WIPI family proteins in vacuolar scission in conjunction with the CSC/Retromer. However, there are several concerns that need to be addressed.

1. The authors show that deletion of vps26 in yeast affects the interaction of Atg18 with Vps35 and come to the conclusion that Vps26 is required for the interaction with the CSC. However, one wonders whether deletion of any subunit (vps35, vps29) of the CSC would impair the interaction since it is unlikely that the remaining individual CSC subunits are capable of associating with each other to form an interaction interface for Atg18. ALso, they should demonstrate biochemically that WIPI1 interacts with the Retromer in mammalian cells beyond showing co-localization by IF.

2. It is unclear why they authors include the Atg18 S57E mutants in the analyses shown in Figure 5 as this mutant is not expressed. I would suggest removing it.

3. There are numerous instances where the bar graphs shown should be replaced with actual data points from the experiments performed. For example: Fig 3a, 5c, 7b, 9b, 10b, and SFig1b.

4. It is interesting that the authors decided to examine the recycling of Transferring Receptor as it is not a known Retromer cargo. Since the authors have gone to extensive lengths to link Atg18/WIPI proteins to CSC/Retromer, they should at a minimum examine the impact of WIPI1 deletion on the recycling of a well known Retromer cargo such as beta2Adrenergic Receptor or Glut1 for example. The data shown that loss of WIPI1 or re-expression of WIPI1 S69 mutants impairs TfnR recycling indicates that the affect of WIPI1 loss are more broadly impacting the recycling of cargoes from the endosomal system. This does not detract from their findings, but I feel they should look at a bonafide Retromer cargo and perform a recycling assay.

5. Similar to point 4 above, it would seem that the authors have a missed opportunity to demonstrate that WIPI also interacts with Retriever and facilitates the recycling of a Retriever cargo (e.g., integrin aV/b1, LDLR, etc...). It would further support the idea that WIPI1 is working as a scission molecule at multiple sites producing endosomal transport carriers.

Referee #3:

This manuscript addresses the interaction between the retromer CSC complex and Atg18 or its mammalian homolog, WIPI1, and the potential role in fission. Most of the study is done in yeast, beginning with the identification of the retromer CSC (but not the affiliated SNX proteins) as an interaction partner for Atg18. Some evidence is provided in semi-purified systems that the yeast vps5/vps17 SNX proteins compete with the retromer CSC for binding to Atg18, although the molecular mechanistics of these interactions have not been fully elucidated. A binding mutant of Atg18 was identified (T56E) which may show decreased binding to the retromer CSC complex, and impaired fission of endosomes was observed in yeast with the Atg18 T56E mutant.

Concerns:
1) The major new finding in this manuscript is the interaction between the retromer CSC and Atg18/WIPI1. A previous paper has already described a role for WIPI1 in fission at the endosome, and this has been characterized fairly extensively, with recycling endosomes in particular. Tubulation has already been noted upon WIP1 knockdown, so the mammalian experiments do not shed significant new light on the function of these proteins. The newer information provided in this manuscript on connecting it with the retromer and the role of this interaction has not been as well defined.

2) Given the differences that have been observed between the yeast and mammalian retromer complexes, it may be too preliminary to infer functions from the yeast system on mammalian systems without significant additional experimentation. While the studies in yeast are generally compelling, the mammalian cell experiments do not especially strengthen the case for the retromer/WIP1 role in fission.

3) The molecular interactions have not been clearly elucidated. In particular, determining which retromer CSC subunit interacts with the Atg18 would be important to identify, and likely fairly simple to do, especially in the yeast. This in turn would allow defining whether the same mammalian CSC subunit(s) bind.

4) While the findings are suggestive of a role for fission at retromer-containing buds from endosomes, there is little effort at reconciling and integrating the known players in endosomal fission with Atg18/WIPI1. Although this reviewer recognizes the necessity for reductionist studies to identify the players in the system, nonetheless some minimal attempt should be made to determine the placement of WIPI1-retromer CSC interactions in the context of the known endosomal fission machinery. For example, how does Arp2/3-mediated actin polymerization enter the equation? Does the retromer simultaneously bring the WASH complex and actin polymerization into action with WIPI1? Do these machineries coordinate and/or compete? What about the proposed role for the ER in fission? Are the tubular endosomes observed the same as those that contain Rab10, EHD1, EHBP1 and MICAL-L1?

5) The data presented in Fig. 9A-B is not convincing. The representative image does not show different patterns of localization for S69A and S69E compared to the WT WIPI1, and there is no control to ensure equal amounts of the GFPVPS26 were transfected. Why is an over-expressed VPS26 used, in any case? There are certainly antibodies available against the endogenous protein. In addition, a Pearson's or correlation coefficient will have trouble distinguishing real overlap in these images, since the pattern of staining is diffuse rather than discrete. Same concern exists for supplemental Fig. 1.

6) There is no quantification of the data exhibited in Fig. 9D. This makes it difficult to accept the subtitle in the legend maintaining that there is an "additive effect" of knocking down VPS35 and expressing mutant WIPI1.

7) The experiments using transferrin to assess recycling lack essential controls. First, normal cells without WIPI1 KO and without transfection need to be used for comparison. Second, a non-relevant GFP (GFP alone or fused to a non-relevant protein) needs to be expressed as a negative control that doesn't affect recycling. In some cases the transfection reagent itself can alter membranes and dynamics of recycling. Third, and very significantly, the authors do not show that the cells begin with 1) equal amounts of internalized transferrin before they measure recycling, and 2) that there are equal levels of transferrin receptor expressed on the plasma membrane for all samples. Same concern exists for Supplemental Fig. 2.

8) The ratio of membrane-associated to soluble Atg18 (and mutant Atg18) has not been quantified (Fig. 7).

9) Why is a quantification of Vps35 in the pellet provided in Fig. 7b, but not quantification of Vps26? To this reviewer it appears as though the Vps26 displays considerable membrane localization in the presence of the Atg18FGGG mutant, unlike the Vps35. This is difficult to reconcile with the statement on p. 8 of the Results where it is noted that "CSC by itself also interacted poorly with vesicles. It was efficiently recruited to them through Atg18WT, but much less through Atg18T56E and ATG18FGGG." Also, it is unclear which band is marked by GFP-Vps29 and which by the ATG18. Does the GFP-Vps29 run more slowly in the supernatant than the pellet?

We thank the referees for their helpful and well-taken comments and suggestions. We considered most of them and integrated them into the revised manuscript, except for a view, which posed very interesting questions but went beyond what can be resolved in the framework of this manuscript. Below, we present the comments of the reviewers in bold type and our response in roman type.

Reviewer #1:

The paper by T. Courtellemont et al. proposes an interesting concept of the formation of a retromer-PROPPIN complex with a function in membrane fission at endosomes and this is poorly studied and understood. It seems rather unnecessary to give this complex a new name to try and own it. Retromer makes many complexes and they do not have separate names.
That's right. But it is not a question of owning a complex. Describing the Vps26/Vps35/Vps29/Atg18 complex by an acronym makes it simply easier to talk and write about it. It also facilitates to find information about the complex for non-specialists in the field, particularly when additional information from other organisms may become available in the future. Without a name for the complex divergent gene nomenclatures will render it difficult to grasp that one talks about corresponding complexes in different organisms (e.g. there is big divergence in the nomenclature of PROPPINs in different organisms). These practical reasons, and the fact that CROP performs a function that is distinct from the well-established retromer function in cargo collection, were our motivations for creating an acronym.
I have one major problem with this work that I believe must be addressed and a significant number of experimental issues cleared up before this paper should be considered for publication in EMBO for which it would likely be suitable if the authors' answers are acceptable.
The reviewer has unfortunately not specified which of the comments addresses the major problem that has been perceived.

In its early days, retromer in yeast was regarded as a heteropetamer but this is historical and not correct. On a gel of yeast retromer there is as much of the sorting nexin SNX3 homologue Grd19p as there is of SNXBARs. Yeast retromer CSC is also able to form membrane attached assemblies with just Grd19p/SNX3 alone (Ungermann and Briggs+Owen labs). Nowhere in this manuscript is Grd19p studied and its role absolutely must be because it is critical to the main message of this work in that SNX BARs compete with Proppins but do they compete with Grd19p/SNX3?

In the first place was Grd19p/SNX3 found in any SILAC experiments? What happens to the proppin+retromer complexes if you add excess Grd19p/SNX3 rather than SNX BARS?
Several observations argue against the notion that the activity of CROP could depend on associated Snx3:

- Vacuole fission, which depends on CROP, is not affected by deletion of SNX3 (now shown in the new Fig. EV1).
- We prepare CSC out of strains overexpressing this complex, but not Snx3. MS analysis of the CSC preparation did not provide any signals for Snx3. Furthermore, Snx3 was not detected in the SILAC experiment that identified Vps26/29/35 as Atg18 interactors (Fig. 1).

This makes rather unlikely that Snx3 has a crucial role for CROP activity and led us to not characterize Snx3 any further. The comment of the reviewer made us recognize that this is a question that should be addressed in the manuscript. In the revised manuscript, we do this in the Results section, provide the complete MS analysis of the SILAC experiment and the CSC preparation as supplementary data on a data repository, and show that snx3Δ cells fragment their vacuoles normally.

Lines 160-170 What happens in VPS29 and VPS35 delete cells, as the text say these are made and tested, on ATG18YFP immunoabsorption

vps29Δ cells do not show a strong effect on the Co-IP of Atg18YFP with Vps26yomCherry. vps35Δ cells to show a significant reduction, which is between the levels seen with vps26Δ and vps29Δ. This data is now shown in the modified Fig. 1.

T56E is important to this work its yield is the same according to the authors but its fold should be checked - a simple CD would suffice - solving the structure of the mutant is unnecessary

We agree. The new Figure EV4 presents CD spectra and melting curves of Atg18wt and atg18T56E. Both proteins show similar behaviour in these assays. Both proteins also bind to liposomes similarly and in a phophoinositide-dependent manner. As a further criterion, we tested the activity of atg18T56E in autophagy in vivo. Cells expressing atg18T56E recover approx. 70% of the autophagic activity of cells expressing ATG18wt. This suggests that atg18T56E is largely functional in vivo. This is also in line with our earlier observations that the membrane fission and the autophagic functions of Atg18 and WIPI1 rely on distinct molecular features (De Leo et al., 2021; Gopaldass et al., 2017).

TfR is not meant to be retromer dependent in its transport and to come from early/recycling endosomes. This would suggest WIPI1 is a

general fusion machine not particularly specific for retromer but functioning anywhere on the endocytic system - could the authors comment please

We do think that WIPI1 is relevant for multiple endosomal trafficking routes. This notion has already been presented in our initial paper on the fission activity of WIPI1 (De Leo et al., 2021), which shows effects of mutations in the hydrophobic loop of WIPI1 on three different endosomal protein exit pathways: Tf recycling, transfer of EGFR to lysosomes, and the exit of Shiga toxin towards the Golgi. For this reason, we had not put particular emphasis on the analysis of a further trafficking route depending on retromer. But, given that we now present Atg18 in the context of a CSC-containing complex, we agree with the referees' comments that adding a retromer-dependent cargo to this list makes sense. In the revised manuscript, we have added experiments on the SNX27-dependent retromer cargo GLUT1, which is strongly depleted from the surface and accumulates intracellularly when the CROP subunit WIPI1 is ablated (new Fig. EV2). By contrast, we found no significant impact of WIPI1 ablation on the distribution of the retriever cargo integrin ß1 (new Fig. EV3), suggesting that not all endosomal traffic is affected.

Discussion - please could the authors explain how they envisage retromer CSC to drive tubule scission when recruited by proppins but not when recruited by SNXBARs when the methods of recruitment, since they compete with each other, would presumably be expected to be the same?

We do not think that retromer CSC is the primary agent in scission but assume that it potentiates the basal fission activity that is provided by Atg18. Our current working hypothesis is that CSC might change the angle at which Atg18 interacts with the membrane and/or the degree to which it can insert its hydrophobic loop into the membrane. Such factors influence the capacity of membrane-inserting proteins to bend or destabilize membranes. Essentially, CSC may scaffold Atg18 on the membrane to potentiate its activity and affect its lipid specificity. We have added a few sentences to the discussion to express this point of view.

Technical questions:

Atg18 forms a complex with Vps26/29/35

As the authors pointed out, a retromer-containing coat can be extracted from yeast. However, the formation of the retromer-Vps5/Vps17 complex requires two types of protein-protein interactions: first, between unstructured N-term part of Vps5 and Vps29 (Seaman and Williams, 2002, Collins et al., 2005) and the second interaction between loops of Vps26 and BAR-domain of Vps5

that was observed in CryoEM structure of the retromer-Vps5 complex (Kovtun et al., 2018). The mass spectrometry analysis results suggest that complex assembly is affected when an Atg18-retromer complex is formed. The authors suggest competing interactions between Vps5 and Atg18 for Vps26 (and possibly Vps35), could authors suggest how Atg18 might affect the interaction between Vps5/Vps29 since they claim complex formation is completely disrupted.
Our IPs and the competition experiments on GUVs do not suggest a complete disruption of the CSC-SNX association, but rather a labilization. Labilization might result from competition of Atg18 even when only one of the two interaction sites of CSC with the SNXs were affected. In addition, Atg18 may also use another binding site that is independent of the binding sites for Vps5/17. Labilization might then result from steric hindrance or allosteric effects. An identification and characterization of the interaction sites should be attempted in the future, but this is a substantial project that could hardly be approached in a satisfactory manner in the framework of the current manuscript. At present, we have no reliable information about the Atg18 binding site and hence prefer not to speculate about it.

Fig1C It looks like there are at least two different yomCherry -Vps5 with almost 10kDa weight difference. Could you please comment on this - why?
We have not pursued this effect because our study is not focused on Vps5. Therefore, we cannot offer any information on this point.

Atg18 and sorting nexins compete for binding to Vps26/29/35
It needs to be clarified what kind of spectroscopy or microscopy methods were used to assess the change of fluorophore properties in the following passage: "To assay formation of CROP directly, we purified Atg18 from bacteria and CSC from yeast, where the Vps29 was labeled with GFP. We noted that the addition of Atg18 to CSC-GFP induced a strong shift in the fluorescence signal of the GFP. Ligand binding can change the properties of protein-bound fluorophores via changes in orientation, contacts, or rotational freedom of the fluorophore. Such changes are a common tool used in titration experiments to determine binding constants."
The method had been described in the Methods section. We had noticed that the fluorescence emission of CSC-GFP at 525 nm (excitation at 488 nm) increases significantly upon incubation with Atg18. This gain in signal could hence be used to assay the binding event. In the revised version, we introduced a sentence in the Results section to mention this.

In Fig. 4, positions a and b need to be swapped as b get mentioned in the text first.

The order of the panels has been adapted accordingly.

It is also puzzling that there is an absence of the signal for CSC-GFP when SNX dimer was added to the CSC-GFP/Atg18 complex. As the authors mentioned previously, retromer can form a stable complex with SNXs. So it would be expected that the mixture loaded into the native gel would contain combinations of different species; showing up as possibly smearing (similar to that observed for ATG18 in the green channel). It would also be essential to include CSC-GFP/SNX complex as a control.
In the native gel system that we employed, SNX gives only a very faint smear, and it appears that the majority of the complex does not even enter the gel. This may explain why CSC essentially disappears from the gel once we add SNX. The reviewer's comment made us realize that the system allows to demonstrate CROP, but that it is unsuited to demonstrate competition by SNX. Therefore, we removed the lane containing SNX and adapted the text accordingly.

Line 203:"Purified CSC forms two major bands, in line with previous observations of monomers and dimers (Kendall et al, 2020)." On the gel, CSC-GFP/Atg18 mix doesn't have an upper (suggested dimer) band. Does it mean the addition of Atg18 affects dimerization of the trimer to form the arches as seen by the Briggs lab? Regardless the Briggs lab paper should be referenced
That would be interesting but we don't think that our experiment permits any conclusions on this. CROP occurs in two bands and the more slowly migrating one contains a higher Atg18 signal than the faster migrating one. This suggests that this higher band might represent a complex with two Atg18 and one CSC. This could be possible because Atg18 alone can oligomerize. In addition, Atg18 affects the most slowly migrating band of CSC, which we tentatively assigned to a CSC dimer. This band becomes significantly weaker, but the lost signal is not recovered in the form of the faster migrating monomer. A possible explanation is that Atg18 might favor the formation of higher-order oligomers, which cannot enter the gel anymore. In order to resolve these possibilities substantial further work will be necessary.

In general it is important to have a real protein ladder for SDS-gels and Western blots visible and not just random numbers added often in relatively different positions.
It is not clear to us what problem the reviewer sees here. Numbers indicating MW have not been placed randomly but represent the position of molecular weight markers on the nitrocellulose, which had been marked by a pen. The numbers locate the position of the MW marker, which anyways

is only an approximate indicator of MW, with sufficient precision to orient us on the gel. We have now added lines next to the numbers to locate the markers more precisely.

In Fig7a the whole image of the gel needs to be shown, possibly in supplementary, as it looks like there are some problems with the background if it was a combination of two different gels in should be clearly stated.
Fig. 7 now shows an entire gel. There has indeed been a problem with the image of the gel, perhaps due to the file conversion. The gel had been cut to remove lines in the middle, which contained samples that are unrelated to the questions treated in this manuscript. The figure has been re-created and the circumference of the gel is now marked by black lines.

There are no secondary antibodies stated nor methods of visualisation
These have now been to the Methods section.

CROP is required for protein exit from mammalian endosomes
"Their abundance and size were further increased by simultaneous knockdown of hVps35" Are these tubules SNX-positive please show?
SNX1 and SNX6 are present on these tubules. However, we do not find them all along the tubules but concentrated in dots that appear to be associated with the tubules. This resembles observations we made for Tf in a previous study on WIPI1 (De Leo et al., 2021). Tf also concentrates in blobs along the tubules and these blobs are continuous with the tubules. The nature of these blobs is intriguing, but we feel that a characterisation of the behavior of the SNXs on these tubules would make the manuscript lose focus and goes beyond its scope.

Discussion
"The current models suggest a two-layered coat, in which an inner layer of SNX proteins recruits a peripheral layer of arch-shaped CSC complexes." This statement needs to be clarified. The two-layer coat was observed only for SNX-BAR-retromer coat (Kovtun et al., 2018), in the case of SNX3-retromer coat, CSC is attached directly to the membrane with the help of cargo and PX-domain of SNX3 (Leneva et al., 2021). In both proposed models dimerization of Vps26 plays an important role, how does it fit into suggested model of interaction Vps26/Atg18? What part of Vps26 (the same loops as in case of Vps5) might be involved in interaction?
Yes, we had neglected the SNX3-containing containing coat in our discussion. This has been corrected and we now describe current knowledge more correctly, considering the direct interaction of Vps26 with

the membrane. At this point, we have absolutely no basis for guessing on the potential interaction sites between Atg18 and Vps26 and/or Vps35. Given that the observed competition effects might also be of allosteric nature, we preferred not to speculate about it. We did, however, add a few phrases to the discussion to illustrate this limitation.

M&M
No secondary antibodies or visualisation methods are described.
No information on MW protein ladders in included.
This information has been added.

Referee #2:

In this manuscript, Mayer and colleagues define an interaction of the CSC (Retromer) with Atg18 (WIPI)that is required for vacuole scission in yeast and scission of endosomal transport carriers in mammalian cells. They term this new complex, the CROP (acronym not defined). They further define that sorting nexin vps17 impairs the recruitment and scission activity of Atg18. Moreover, they demonstrate that a highly conserved serine residue found in both Atg18 and WIPI proteins is involved in interaction of Atg18 with the CSC, and hence, scission of the yeast-like vacuole, GUVs and mammalian endosomes. Overall, this is a very interesting paper with highly supportive data that makes an important discovery regarding the role of Atg18/WIPI family proteins in vacuolar scission in conjunction with the CSC/Retromer. However, there are several concerns that need to be addressed.

1. The authors show that deletion of vps26 in yeast affects the interaction of Atg18 with Vps35 and come to the conclusion that Vps26 is required for the interaction with the CSC. However, one wonders whether deletion of any subunit (vps35, vps29) of the CSC would impair the interaction since it is unlikely that the remaining individual CSC subunits are capable of associating with each other to form an interaction interface for Atg18.

The new Fig. 1 now shows also co-adsorption experiments from vps29Δ and vps35Δ cells, where Vps26yomCherry was pulled down. vps35Δ labilizes the Vps26/Atg18 interaction, whereas vps29Δ has no significant effect.

ALso, they should demonstrate biochemically that WIPI1 interacts with the Retromer in mammalian cells beyond showing co-localization by IF.

We now show adsorption of mammalian retromer to immobilized WIPI1. in the new Fig. EV5. It should be noted that in our hands WIPI1 is much less well behaved than Atg18, so similar biochemical experiments as we showed for yeast CROP are not yet possible for us.

2. It is unclear why they authors include the Atg18 S57E mutants in the analyses shown in Figure 5 as this mutant is not expressed. I would suggest removing it.

We systematically made A and E substitutions for all the residues that we analyzed. Simply leaving out the S57E mutant would provoke the question why we didn't try this substitution. Therefore, we preferred to leave it in the figure to show that this substitution cannot be analyzed because it is not expressed.

3. There are numerous instances where the bar graphs shown should be replaced with actual data points from the experiments performed. For example: Fig 3a, 5c, 7b, 9b, 10b, and SFig1b.
We have inserted individual data points into the revised figures.

4. It is interesting that the authors decided to examine the recycling of Transferring Receptor as it is not a known Retromer cargo. Since the authors have gone to extensive lengths to link Atg18/WIPI proteins to CSC/Retromer, they should at a minimum examine the impact of WIPI1 deletion on the recycling of a well known Retromer cargo such as beta2Adrenergic Receptor or Glut1 for example. The data shown that loss of WIPI1 or re-expression of WIPI1 S69 mutants impairs TfnR recycling indicates that the affect of WIPI1 loss are more broadly impacting the recycling of cargoes from the endosomal system. This does not detract from their findings, but I feel they should look at a bonafide Retromer cargo and perform a recycling assay.
We had not arrived at this project by seeking to link Atg18 to retromer but rather by an unbiased search for additional proteins that are required for membrane fission of vacuoles. Hence our initial negligence of actual retromer cargo. But we agree with the reviewer that it makes sense to show it. The new Fig. EV2 presents a strong accumulation of GLUT1 on intracellular compartments of WIPI1 knockouts and its depletion from the cell surface. Together with our previous data this suggests that WIPI1 has a broad impact on the exit of a variety of cargos from endosomes. The block is not general, however, because integrin ß1 shows no comparable change in localisation in WIPI1KO cells (see new Fig. EV3).

5. Similar to point 4 above, it would seem that the authors have a missed opportunity to demonstrate that WIPI also interacts with Retriever and facilitates the recycling of a Retriever cargo (e.g., integrin aV/b1, LDLR, etc...). It would further support the idea that WIPI1 is working as a scission molecule at multiple sites producing endosomal transport carriers.
We have analyzed the effect on integrin ß1 (new Fig. EV3). Its distribution between the interior of the cell and the plasma membrane was not significantly affected by knockout of WIPI1. However, as we show in the revised version and our previous manuscript (De Leo, 2021), WIPI1 impairs endosomal exit of GLUT1, TfR, EGFR, and Shiga toxin, suggesting that it functions in multiple but not all sorting pathways from endosomes.

Referee 3:

This manuscript addresses the interaction between the retromer CSC complex and Atg18 or its mammalian homolog, WIPI1, and the potential role in fission. Most of the study is done in yeast, beginning with the identification of the retromer CSC (but not the affiliated SNX proteins) as an interaction partner for Atg18. Some evidence is provided in semi-purified systems that the yeast vps5/vps17 SNX proteins compete with the retromer CSC for binding to Atg18, although the molecular mechanistics of these interactions have not been fully elucidated. A binding mutant of Atg18 was identified (T56E) which may show decreased binding to the retromer CSC complex, and impaired fission of endosomes was observed in yeast with the Atg18 T56E mutant.

Concerns:

1) The major new finding in this manuscript is the interaction between the retromer CSC and Atg18/WIPI1. A previous paper has already described a role for WIPI1 in fission at the endosome, and this has been characterized fairly extensively, with recycling endosomes in particular. Tubulation has already been noted upon WIP1 knockdown, so the mammalian experiments do not shed significant new light on the function of these proteins. The newer information provided in this manuscript on connecting it with the retromer and the role of this interaction has not been as well defined.

This criticism is relatively unspecific. We feel that the identification of a novel complex that potentiates the fission activity of Atg18 or WIPI1 drastically is an important finding. The molecular link between membrane fission, Atg18 and CSC, which this new complex provides, also changes our view on the roles and molecular activities of retromer.

2) Given the differences that have been observed between the yeast and mammalian retromer complexes, it may be too preliminary to infer functions from the yeast system on mammalian systems without significant additional experimentation. While the studies in yeast are generally compelling, the mammalian cell experiments do not especially strengthen the case for the retromer/WIP1 role in fission.

We appreciated that the reviewer finds our work on the yeast complex compelling, because this is the essential core of our study. We had added a few experiments in mammalian cells to illustrate the potential that a similar complex may exist in mammals and that it may provide similar activities in mammalian cells. We agree that similar in vitro experiments as we have done for the yeast complex should be done also for the mammalian system. This is hampered by the fact that in our hands

mammalian WIPI1 is not nearly as well behaved as Atg18 and difficult to express and purify. Presenting a full set of equivalent experiments with mammalian proteins would also bring the manuscript to an excessive volume. To strengthen our point that CROP may also exist in mammalian cells we have added the new Fig. EV5, which shows enrichment of retromer through immobilized WIPI1-HA.

3) The molecular interactions have not been clearly elucidated. In particular, determining which retromer CSC subunit interacts with the Atg18 would be important to identify, and likely fairly simple to do, especially in the yeast. This in turn would allow defining whether the same mammalian CSC subunit(s) bind.
We have analyzed the Atg18-CSC interaction in mutants of all its subunits and show the result in the new Fig. 1. Whereas deletion of VPS35 significantly reduced the interaction of Atg18 with Vps26, deletion of VPS29 had little effect. Thus, both Vps26 and Vps35 appear to contribute to the interaction of Atg18 and CSC. Since bioinformatic approaches have not permitted to guess on potential binding sites on Vps35 and Vps26, a further, detailed characterisation of the binding sites will be a substantial structural project, which could not be approached as an addition to this study.

4) While the findings are suggestive of a role for fission at retromer-containing buds from endosomes, there is little effort at reconciling and integrating the known players in endosomal fission with Atg18/WIPI1. Although this reviewer recognizes the necessity for reductionist studies to identify the players in the system, nonetheless some minimal attempt should be made to determine the placement of WIPI1-retromer CSC interactions in the context of the known endosomal fission machinery. For example, how does Arp2/3-mediated actin polymerization enter the equation? Does the retromer simultaneously bring the WASH complex and actin polymerization into action with WIPI1? Do these machineries coordinate and/or compete? What about the proposed role for the ER in fission? Are the tubular endosomes observed the same as those that contain Rab10, EHD1, EHBP1 and MICAL-L1?
This is a long list of important questions, and we share the reviewer's interest for them. However, addressing each of these questions is experimentally complex and essentially requires a separate study. Although these questions are very relevant and appropriate, it is not possible to resolve them in the framework of this manuscript. We will address some of them in the future. We do acknowledge the importance of other factors for the departure of endosomal carriers, mentioned them in the introduction and underline in the discussion that their relationship to CROP should be

explored in the future. But this is simply not possible in a manuscript that already has 15 figures, and which is primarily focused on the yeast complex.

5) The data presented in Fig. 9A-B is not convincing. The representative image does not show different patterns of localization for S69A and S69E compared to the WT WIPI1, and there is no control to ensure equal amounts of the GFPVPS26 were transfected. Why is an over-expressed VPS26 used, in any case? There are certainly antibodies available against the endogenous protein.
Antibodies against the endogenous Vps26 are indeed available. A problem is that the long tubules that accumulate in the WIPI1 mutants are very poorly maintained upon fixation of the cells (we tried numerous protocols). Therefore, we analyze living cells expressing fluorescent protein fusions. We mention this constraint now in the revised version. The fusion proteins are not heavily overexpressed. Their expression levels are not affected by the different WIPI1 variants. and that of the endogenous protein in the different cell lines is now shown in Suppl. Fig. 1.
It is not clear to us why the reviewer finds that the co-localization pattern of S69E would not be significantly different from that of the WT in the presented images. WT shows significant overlap of red and green structures, which S69A does not and S69E to a lesser degree.

In addition, a Pearson's or correlation coefficient will have trouble distinguishing real overlap in these images, since the pattern of staining is diffuse rather than discrete. Same concern exists for supplemental Fig. 1.

We agree with the argument against using the Pearson's coefficient in this context. We have therefore re-analyzed the images that formed the basis of the previous Figs. 9 and S1 (now EV6). The modified procedure is described in detail in the Methods section. Briefly, the tubules in the images were segmented using the Canny or Sobel filter plugin of ImageJ. The resulting masked regions were subjected to thresholding and the co-localisation of pixels in the two channels was quantified through Manders' coefficient M2.

6) There is no quantification of the data exhibited in Fig. 9D. This makes it difficult to accept the subtitle in the legend maintaining that there is an "additive effect" of knocking down VPS35 and expressing mutant WIPI1.
The length of tubules has been measured by manual tracing. It is now shown in the new Fig. 9G.

7) The experiments using transferrin to assess recycling lack essential controls. First, normal cells without WIPI1 KO and without transfection need to be used for comparison. Second, a non-relevant GFP (GFP alone or fused to a non-relevant protein) needs to be expressed as a negative control that doesn't affect recycling. In some cases the transfection reagent itself can alter membranes and dynamics of recycling. Third, and very significantly, the authors do not show that the cells begin with 1) equal amounts of internalized transferrin before they measure recycling, and 2) that there are equal levels of transferrin receptor expressed on the plasma membrane for all samples. Same concern exists for Supplemental Fig. 2.
These concerns are valid, but we have published the suggested controls for this very experiment in our recent paper (De Leo et al., 2021). They show that WIPI1 and the transfection procedure do not impact the loading of cells with transferrin but only its recycling back to the cell surface. We feel that expressing a non-relevant GFP is a much weaker control than showing the fusion to WIPI1-WT because the GFP is most likely to have an impact by sterically interfering with WIPI1 function. The WIPI1-WT-EGFP rescues all discernable phenotypes of a WIPI1 KO or a WIPI1 knockdown (De Leo et al., 2021) and hence is functional. We also show a dominant-negative effect of the WIPI1-eGFP variants, but not of the WT, in Fig. EV7. This implies that the EGFP tag itself does not have detrimental effects on Tf sorting.
The assays in the manuscript that we submit now have been done with the same cell lines and under the same conditions, so it would be unjustifiable to publish the same controls again. We have hence inserted a phrase in the Results section to refer the reader to these already published results. It should also be noted that Fig. 10 of our submitted manuscript contains, in the same fields, cells that have all undergone the same transfection procedure but some of which are not expressing the WIPI1 variants. They provide an internal control for non-transfected cells.

8) The ratio of membrane-associated to soluble Atg18 (and mutant Atg18) has not been quantified (Fig. 7).
This quantification has now been added.

9) Why is a quantification of Vps35 in the pellet provided in Fig. 7b, but not quantification of Vps26? To this reviewer it appears as though the Vps26 displays considerable membrane localization in the presence of the Atg18FGGG mutant, unlike the Vps35. This is difficult to reconcile with the statement on p. 8 of the Results where it is noted that "CSC by itself also interacted poorly with vesicles. It was efficiently recruited to them through Atg18WT, but much less through Atg18T56E and ATG18FGGG." Also, it is unclear which band is

marked by GFP-Vps29 and which by the ATG18. Does the GFP-Vps29 run more slowly in the supernatant than the pellet?

We consider Vps35 a more meaningful marker for recruitment of CSC than Vps26. Vps26 has membrane-inserting activity itself (Leneva et al., 2021) and can therefore have an Atg18-independent affinity for membranes. We assume that this is the reason for the Atg18-independent fractionation of Vps26 with the liposomes. This Atg18-independent fraction does not co-recruit comparable amounts of Vps35 and Vps29, suggesting that it represents free Vps26 that is not incorporated in CSC. We acknowledge that this behaviour of Vps26 should be commented on in the paper and have done this in the revised version.

Regarding the behaviour of CSC in the GUV experiments it should be considered that here we detect Vps29-GFP. A potential background of non-fluorescent, solitary Vps26 would not be detected in this experiment. Also, the lipid composition of the GUVs is not the same as that of the SUVs. In the revised version, we now underline the difference in the lipid composition of the SUVs and the GUVs.

Concerning GFP-Vps29, Atg18 and the running behavior in the gel: We have now marked the positions of Atg18 and Vps29-GFP by asterisks. The pellets contain significant amounts of lipids, which can influence the migration of proteins in the lane. Therefore, we prefer not to interpret or discuss differences in running behavior between proteins in the supernatant and the pellet.

1st Revision - Editorial Decision 1st Mar 2022

Dear Prof. Mayer,

Thank you for the submission of your revised manuscript to The EMBO Journal. Two of the original referees have looked at the paper and now consider that you have properly dealt with all of their major concerns. Referee #1 requires some further discussion on the potential role of Snx3/Grd19. Additionally, before we can proceed with the acceptance of your study, there are a few minor editorial issues that need your attention:

- Please provide 3 to 5 keywords describing the topic of your paper.
- The manuscript needs a Data Availability Section where you describe the databases in which datasets have been made available. For transparency, this section is required even if just to mention that there are no public datasets associated to the results in the paper.
- You need to provide a "Disclosure and Competing Interests Statement".
- While you provided funding information to the manuscript handling system online, please add it to the manuscript as well.
- Figures must be uploaded as individual files and removed from the main manuscript.
- Please correct the position of one of the scale bars in Figure 7D.
- Supplementary information should be renamed as Appendix and should be presented as a single file starting with a Table of Contents. Correct nomenclature for Appendix figures and tables is "Appendix Figure S1" or "Appendix Table S1" respectively. Please rename "Supplemental Table 1" as "Table 1" or "Appendix Table 1", including it in the Appendix file in the latter case. Modify manuscripts callouts accordingly.
- Please provide callouts in the manuscript for Figure 10 panels, EV figures and Supplemental Figure 1, which as explained above should be renamed Appendix Figure S1.

Please provide the synopsis files. The paper's synopsis is composed of:
- a short 'blurb' text summarizing in two sentences the study (max. 250 characters) and three to four 'bullet points' highlighting the main findings. Bullet points and standfirst text should be submitted as a separate manuscript file in LaTeX, RTF or MS Word format. This text has not been provided.
- A "synopsis image", which can be used as a "visual title" for the synopsis section of your paper. While you provide this image, it needs to be resized. This figure must be in PNG or JPG format with pixel dimensions of 550 x 300-600 (width x height).

Find also attached a word document with some comments to modify the figure legends from our data editor at the production team. Please, provide the information requested.

Let me know if you have any further questions regarding any of these points. Thank you again for giving us the chance to consider your manuscript for The EMBO Journal and congratulations!

I look forward to receiving the final version of your manuscript with these minor changes included.

Yours sincerely,

David del Alamo
Editor
The EMBO Journal

Further information is available in our Guide For Authors: https://www.embopress.org/page/journal/14602075/authorguide

Please click on the link below to submit the revision online:

Link Not Available

Referee #1:
The authors have addressed most of my issues, although I still believe that a further investigation on Snx3/Grd19p would increase the interest and scope of the paper. As the authors have stated that they do not believe Snx3/Grd19 is involved in CROP induced scission of retromer tubes they consider are dependent on use of SNX BARs then at least all reference to SNX in the abstract should be changed to SNXBARs as there is no evidence presented other than inferred negative data, that SNX3 is involved. SNX3 is clearly at least as, if not more important, than SNXBARs in retromer based trafficking in mammalian cells

although its relative contribution in yeast is uncharacterised and if the authors choose to ignore this then they should not make claims for all SNXs in the abstract. I also feel a rather more complete explanation of why they have chosen to ignore Snx3/Grd19 despite its obvious importance should be more fully explained rather than the rather cursory attempt currently present. Such an explanation should include how retromer coated tubules lacking SNXBARs but including SNX3/Grd19 switch from cargo acquisition and formation to scission if CROP is not involved.

Referee #2:

The authors have made significant effort to respond to my original critique, which I feel has further supported their study. I support publication of this manuscript in The EMBO Journal.

Rebuttal

We thank reviewer 1 for her/his interest in our findings and want to briefly come back to the reviewer's arguments concerning SNX3. We have understood SNX in the abstract as referring to the yeast SNX complex but recognize now that this is potentially ambiguous. We hence followed the recommendation of the reviewer to use SNXBAR instead of just SNX.

We also want to emphasize that we really appreciate this reviewer's constructive comments, which were very helpful for us, and the reviewer's concern for the impact of our manuscript. If we avoided to fully address the SNX3 issue in this manuscript it is not out of lack of interest, nor because we would not recognize its relevance. An honest reason is that we do work on fission of SNX3-coated carriers and that we have a set of data on it, which is, however, too voluminous to add it to the current manuscript. We hope to publish it as a separate piece of work soon. In our view, not addressing SNX3/Grd19 in the current manuscript is not a gross shortcoming because it is focused on the identification of CROP and not on a characterization of its role in the full scale of endo-lysosomal trafficking pathways. Therefore, we had decided to analyze only a few examples for endosomal protein trafficking routes, but not to test the full spectrum of known protein exit mechanisms. SNX3 is not the only one that we left out. We agree that this and numerous other aspects of endo-lysosomal trafficking remain to be analyzed with respect to the role of CROP and we hope to fill some of these holes in the future.

Dear Prof. Mayer,

I am pleased to inform you that your manuscript has been accepted for publication in the EMBO Journal.

Ther is just one minor issue that still needs to be solved before we proceed with the production phase: callouts for figures EV2-5 are missing in the text and need to be included. Please send us a revised version of the text and we will upload it for you.

Please note that it is EMBO Journal policy for the transcript of the editorial process (containing referee reports and your response letter) to be published as an online supplement to each paper. If you do NOT want this, you will need to inform the Editorial Office via email immediately. More information is available here:
https://www.embopress.org/page/journal/14602075/authorguide#transparentprocess

Your manuscript will be processed for publication in the journal by EMBO Press. Manuscripts in the PDF and electronic editions of The EMBO Journal will be copy edited, and you will be provided with page proofs prior to publication. Please note that supplementary information is not included in the proofs.

Please note that you will be contacted by Wiley Author Services to complete licensing and payment information. The required 'Page Charges Authorization Form' is available here: https://www.embopress.org/pb-assets/embo-site/tej_apc.pdf

Should you be planning a Press Release on your article, please get in contact with embojournal@wiley.com as early as possible, in order to coordinate publication and release dates.

If you have any questions, please do not hesitate to call or email the Editorial Office. Thank you for your contribution to The EMBO Journal.

Yours sincerely,

David del Alamo
Editor
The EMBO Journal

EMBO Press Author Checklist

Corresponding Author Name: Andreas Mayer

Journal Submitted to: EMBO J
Manuscript Number: EMBOJ-2021-109646R

USEFUL LINKS FOR COMPLETING THIS FORM
The EMBO Journal - Author Guidelines
EMBO Reports - Author Guidelines
Molecular Systems Biology - Author Guidelines
EMBO Molecular Medicine - Author Guidelines

Reporting Checklist for Life Science Articles (updated January 2022)
This checklist is adapted from Materials Design Analysis Reporting (MDAR) Checklist for Authors. MDAR establishes a minimum set of requirements in transparent reporting in the life sciences (see Statement of Task: 10.31222/osf.io/9sm4x). Please follow the journal's guidelines in preparing your manuscript.
Please note that a copy of this checklist will be published alongside your article.

Abridged guidelines for figures
1. Data
The data shown in figures should satisfy the following conditions:
- the data were obtained and processed according to the field's best practice and are presented to reflect the results of the experiments in an accurate and unbiased manner.
- ideally, figure panels should include only measurements that are directly comparable to each other and obtained with the same assay.
- plots include clearly labeled error bars for independent experiments and sample sizes. Unless justified, error bars should not be shown for technical replicates.
- if n<5, the individual data points from each experiment should be plotted. Any statistical test employed should be justified.
- Source Data should be included to report the data underlying figures according to the guidelines set out in the authorship guidelines on Data Presentation.

2. Captions
Each figure caption should contain the following information, for each panel where they are relevant:
- a specification of the experimental system investigated (eg cell line, species name).
- the assay(s) and method(s) used to carry out the reported observations and measurements.
- an explicit mention of the biological and chemical entity(ies) that are being measured.
- an explicit mention of the biological and chemical entity(ies) that are altered/varied/perturbed in a controlled manner.
- the exact sample size (n) for each experimental group/condition, given as a number, not a range;
- a description of the sample collection allowing the reader to understand whether the samples represent technical or biological replicates (including how many animals, litters, cultures, etc.).
- a statement of how many times the experiment shown was independently replicated in the laboratory.
- definitions of statistical methods and measures:
  - common tests, such as t-test (please specify whether paired vs. unpaired), simple χ2 tests, Wilcoxon and Mann-Whitney tests, can be unambiguously identified by name only, but more complex techniques should be described in the methods section;
  - are tests one-sided or two-sided?
  - are there adjustments for multiple comparisons?
  - exact statistical test results, e.g., P values = x but not P values < x;
  - definition of 'center values' as median or average;
  - definition of error bars as s.d. or s.e.m.

Please complete ALL of the questions below.
Select "Not Applicable" only when the requested information is not relevant for your study.

Materials

Newly Created Materials	Information included in the manuscript?	In which section is the information available? (Reagents and Tools Table, Materials and Methods, Figures, Data Availability Section)
New materials and reagents need to be available; do any restrictions apply?	Yes	Methods

Antibodies	Information included in the manuscript?	In which section is the information available? (Reagents and Tools Table, Materials and Methods, Figures, Data Availability Section)
For **antibodies** provide the following information:<br>- Commercial antibodies: RRID (if possible) or supplier name, catalogue number and or/clone number<br>- Non-commercial: RRID or citation	Yes	Methods

DNA and RNA sequences	Information included in the manuscript?	In which section is the information available? (Reagents and Tools Table, Materials and Methods, Figures, Data Availability Section)
**Short novel DNA or RNA including primers, probes**: provide the sequences.	Yes	Table 1

Cell materials	Information included in the manuscript?	In which section is the information available? (Reagents and Tools Table, Materials and Methods, Figures, Data Availability Section)
**Cell lines:** Provide species information, strain. Provide accession number in repository **OR** supplier name, catalog number, clone number, and/**OR** RRID.	Yes	
**Primary cultures:** Provide species, strain, sex of origin, genetic modification status.	Not Applicable	
Report if the cell lines were recently **authenticated** (e.g., by STR profiling) and tested for mycoplasma contamination.	Not Applicable	

Experimental animals	Information included in the manuscript?	In which section is the information available? (Reagents and Tools Table, Materials and Methods, Figures, Data Availability Section)
**Laboratory animals or Model organisms:** Provide species, strain, sex, age, genetic modification status. Provide accession number in repository **OR** supplier name, catalog number, clone number, **OR** RRID.	Yes	Methods
**Animal observed in or captured from the field:** Provide species, sex, and age where possible.	Not Applicable	
Please detail **housing and husbandry conditions**.	Not Applicable	

Plants and microbes	Information included in the manuscript?	In which section is the information available? (Reagents and Tools Table, Materials and Methods, Figures, Data Availability Section)
**Plants:** provide species and strain, ecotype and cultivar where relevant, unique accession number if available, and source (including location for collected wild specimens).	Not Applicable	
**Microbes:** provide species and strain, unique accession number if available, and source.	Yes	Methods

Human research participants	Information included in the manuscript?	In which section is the information available? (Reagents and Tools Table, Materials and Methods, Figures, Data Availability Section)
If collected and within the bounds of privacy constraints report on age, sex and gender or ethnicity for all study participants.	Not Applicable	

Core facilities	Information included in the manuscript?	In which section is the information available? (Reagents and Tools Table, Materials and Methods, Figures, Data Availability Section)
If your work benefited from core facilities, was their service mentioned in the acknowledgments section?	Yes	

Design

Study protocol	Information included in the manuscript?	In which section is the information available? (Reagents and Tools Table, Materials and Methods, Figures, Data Availability Section)
If study protocol has been **pre-registered, provide DOI in the manuscript**. For clinical trials, provide the trial registration number **OR** cite DOI.	Not Applicable	
Report the **clinical trial registration number** (at ClinicalTrials.gov or equivalent), where applicable.	Not Applicable	

Laboratory protocol	Information included in the manuscript?	In which section is the information available? (Reagents and Tools Table, Materials and Methods, Figures, Data Availability Section)
Provide DOI OR other citation details if **external detailed step-by-step protocols** are available.	Not Applicable	

Experimental study design and statistics	Information included in the manuscript?	In which section is the information available? (Reagents and Tools Table, Materials and Methods, Figures, Data Availability Section)
Include a statement about **sample size** estimate even if no statistical methods were used.	Not Applicable	
Were any steps taken to minimize the effects of subjective bias when allocating animals/samples to treatment (e.g. **randomization procedure**)? If yes, have they been described?	Not Applicable	
Include a statement about **blinding** even if no blinding was done.	Not Applicable	
Describe **inclusion/exclusion criteria** if samples or animals were excluded from the analysis. Were the criteria pre-established? If sample or data points were omitted from analysis, report if this was due to attrition or intentional exclusion and provide justification.	Not Applicable	
For every figure, are **statistical tests** justified as appropriate? Do the data meet the assumptions of the tests (e.g., normal distribution)? Describe any methods used to assess it. Is there an estimate of variation within each group of data? Is the variance similar between the groups that are being statistically compared?	Yes	Methods, Fig. legends

Sample definition and in-laboratory replication	Information included in the manuscript?	In which section is the information available? (Reagents and Tools Table, Materials and Methods, Figures, Data Availability Section)
In the figure legends: state number of times the experiment was **replicated** in laboratory.	Yes	
In the figure legends: define whether data describe **technical or biological replicates**.	Yes	

Ethics

Ethics	Information included in the manuscript?	In which section is the information available? (Reagents and Tools Table, Materials and Methods, Figures, Data Availability Section)
Studies involving **human participants**: State details of **authority granting ethics approval** (IRB or equivalent committee(s), provide reference number for approval.	Not Applicable	
Studies involving **human participants**: Include a statement confirming that **informed consent** was obtained from all subjects and that the experiments conformed to the principles set out in the WMA Declaration of Helsinki and the Department of Health and Human Services Belmont Report.	Not Applicable	
Studies involving **human participants:** For publication of **patient photos**, include a statement confirming that consent to publish was obtained.	Not Applicable	
Studies involving experimental **animals**: State details of **authority granting ethics approva**l (IRB or equivalent committee(s), provide reference number for approval. Include a statement of compliance with ethical regulations.	Not Applicable	
Studies involving **specimen and field samples:** State if relevant **permits** obtained, provide details of authority approving study; if none were required, explain why.	Not Applicable	

Dual Use Research of Concern (DURC)	Information included in the manuscript?	In which section is the information available? (Reagents and Tools Table, Materials and Methods, Figures, Data Availability Section)
Could your study fall under dual use research restrictions? Please check biosecurity documents and list of **select agents and toxins** (CDC): https://www.selectagents.gov/sat/list.htm	Not Applicable	
If you used a select agent, is the security level of the lab appropriate and reported in the manuscript?	Not Applicable	
If a study is subject to dual use research of concern regulations, is the name of the **authority granting approval and reference number** for the regulatory approval provided in the manuscript?	Not Applicable	

Reporting

The MDAR framework recommends adoption of discipline-specific guidelines, established and endorsed through community initiatives. Journals have their own policy about requiring specific guidelines and recommendations to complement MDAR.

Adherence to community standards	Information included in the manuscript?	In which section is the information available? (Reagents and Tools Table, Materials and Methods, Figures, Data Availability Section)
State if relevant guidelines or checklists (e.g., **ICMJE, MIBBI, ARRIVE, PRISMA**) have been followed or provided.	Not Applicable	
For **tumor marker prognostic studies,** we recommend that you follow the **REMARK** reporting guidelines (see link list at top right). See author guidelines, under 'Reporting Guidelines'. Please confirm you have followed these guidelines.	Not Applicable	
For **phase II and III randomized controlled trials**, please refer to the **CONSORT** flow diagram (see link list at top right) and submit the CONSORT checklist (see link list at top right) with your submission. See author guidelines, under 'Reporting Guidelines'. Please confirm you have submitted this list.	Not Applicable	

Data Availability

Data availability	Information included in the manuscript?	In which section is the information available? (Reagents and Tools Table, Materials and Methods, Figures, Data Availability Section)
Have **primary datasets** been deposited according to the journal's guidelines (see 'Data Deposition' section) and the respective accession numbers provided in the Data Availability Section?	Yes	Methods
Were **human clinical and genomic datasets** deposited in a public access-controlled repository in accordance to ethical obligations to the patients and to the applicable consent agreement?	Not Applicable	
Are **computational models** that are central and integral to a study available without restrictions in a machine-readable form? Were the relevant accession numbers or links provided?	Not Applicable	
If publicly available data were reused, provide the respective **data citations in the reference list**.	Not Applicable	
